# Temporal metabolic and transcriptomic characteristics crossing islets and liver reveal dynamic pathophysiology in diet-induced diabetes

**DOI:** 10.1016/j.isci.2021.102265

**Published:** 2021-03-05

**Authors:** Rui Gao, Qi Fu, He-Min Jiang, Min Shen, Rui-Ling Zhao, Yu Qian, Yun-Qiang He, Kuan-Feng Xu, Xin-Yu Xu, Heng Chen, Quan Zhang, Tao Yang

**Affiliations:** 1 Department of Endocrinology and Metabolism, The First Affiliated Hospital of Nanjing Medical University, Nanjing, Jiangsu 210029, China; 2 Oxford Centre for Diabetes, Endocrinology and Metabolism, Radcliffe Department of Medicine, University of Oxford, Oxford OX37LE, UK

**Keywords:** Animal Physiology, Diabetology, Transcriptomics

## Abstract

To investigate the molecular mechanisms underlying islet dysfunction and insulin resistance in diet-induced diabetes, we conducted temporal RNA sequencing of tissues responsible for insulin secretion (islets) and action (liver) every 4 weeks in mice on high-fat (HFD) or chow diet for 24 weeks, linking to longitudinal profile of metabolic characteristics. The diverse responses of α, β, and δ cells to glucose and palmitate indicated HFD-induced dynamic deterioration of islet function from dysregulation to failure. Insulin resistance developed with variable time course in different tissues. Weighted gene co-expression network analysis and Ingenuity Pathway Analysis implicated islets and liver jointly programmed β-cell compensatory adaption via cell proliferation at early phase and irreversible islet dysfunction by inappropriate immune response at later stage, and identified interconnected molecules including growth differentiation factor 15. Frequencies of T cell subpopulation showed an early decrement in Tregs followed by increases in Th1 and Th17 cells during progression to diabetes.

## Highlights

• Diet-induced diabetes is featured by transition from islet dysfunction to failure
• Insulin resistance develops with variable time course in different tissues
• Dynamics of islet and liver molecular network interplay at different stages
• T-cell-mediated immune response participates via priming and amplification phases

## Graphical abstract

**Figure:**
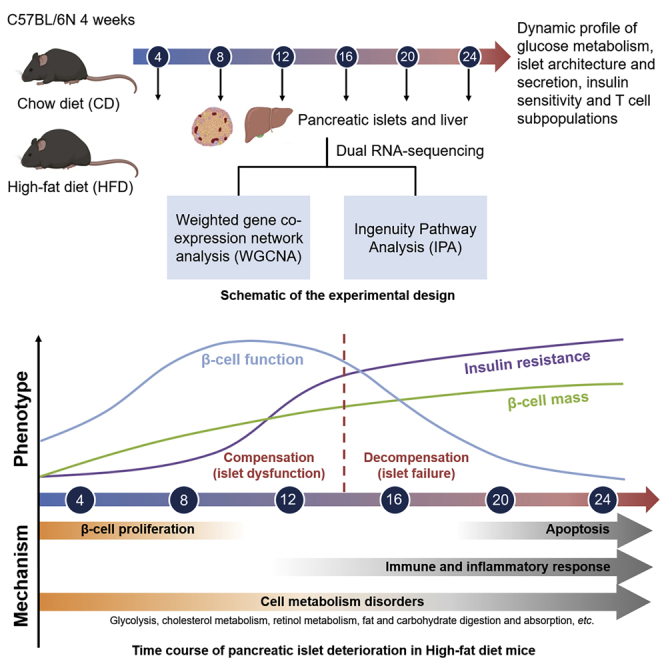

## Introduction

Overnutrition is a major forerunner of type 2 diabetes mellitus (T2DM), which can both enhance secretion of insulin and attenuate its metabolic actions on peripheral tissues including liver, skeletal muscle, and adipose tissue (Czech, 2017). Regardless of whether basal hyperinsulinemia or insulin resistance is the primary driver of T2DM, it is widely accepted that the progressive deterioration of β-cell function/loss of functional β-cell mass is key to the onset of diabetes (Saisho, 2015). In order to identify molecules and mechanisms associated with β-cells’ transition from adaption to failure, high-throughput “omics” technologies (particularly microarray-based transcriptomics and mass-spectrometry-based proteomics) were broadly used on islets from different T2DM animal models (Aga et al., 2020; Hou et al., 2017; Kluth et al., 2014; Neelankal John et al., 2018; Roat et al., 2014) and diabetic human donors (Mencucci et al., 2021). Longitudinal observations and temporal “omics” analyses across the full spectrum of disease have gradually established a comprehensive picture of diabetic progression (Fadista et al., 2014; Hou et al., 2017; Solimena et al., 2018). However, few studies systematically described the metabolic phenotypes and associated the sequencing data with these characteristics. Moreover, because environmental manipulation plays a fundamental role in obesity development, high-fat diet (HFD) is thought to model the human situation of obesity-induced diabetes more accurately than genetic animal models (Hou et al., 2017; Kluth et al., 2014).

Individual β-cell can sense a multitude of signals and change its secretory responses according to metabolic demands. In recent years, endocrine and autocrine/paracrine factors have aroused great interest; however, a limited number of studies concentrated on the inter-organ crosstalk. Exogenous factors, such as humoral and neural signals originating from hepatocytes (El Ouaamari et al., 2013; Imai et al., 2008), adipocytes, and various immune cells, not only constitute a significant link between obesity and insulin resistance but also impact β-cell function and cell mass (Tanabe et al., 2017). Liver, an important organ participating in fat metabolism, glycogen synthesis, and decomposition, may play a role in regulating pancreatic β-cells. We previously identified that hepatocytes derived extracellular vesicles from HFD-induced obese mice could modulate genes expression and promote proliferation of islet β-cells through miRNA (Fu et al., 2019). During the last decade, other liver-derived circulating factors such as hepatic growth factor (HGF) (Alvarez-Perez et al., 2014; Mellado-Gil et al., 2011), leukocyte-neutrophil elastase inhibitor (SerpinB1) (El Ouaamari et al., 2016), kisspeptin (Song et al., 2014), and fibroblast growth factor 21 (FGF21) (Kharitonenkov et al., 2005; Wente et al., 2006) have been reported to directly affect islet secretion, proliferation, and regeneration.

In the present study, experiments were conducted to (1) define the timing of key physiological and molecular events in a rodent model of diet-induced diabetes characterized by hyperinsulinemia and insulin resistance; (2) determine the sequential repertoire of distinct mechanisms that underlie β-cells’ transition from dysfunction to failure; (3) assess the relationship of transcriptomic changes between islets (responsible for hyperinsulinemia) and liver (involved in insulin resistance), and (4) determine the potential interconnected genes participating in this crosstalk. Thus, we established a dynamic profile of glucose metabolism, islet architecture and secretion, systemic and tissue-specific insulin resistance, and T cell subpopulations in diet-treated C57BL/6N mice over a time course of 24 weeks. Subsequent transcriptomic analyses of islets and liver unveiled the chronological order of molecular events during the deterioration of pancreatic islet function.

## Results

### Experimental design and general metabolic characteristics of HFD in C57BL/6N mice

**Figure 1:**
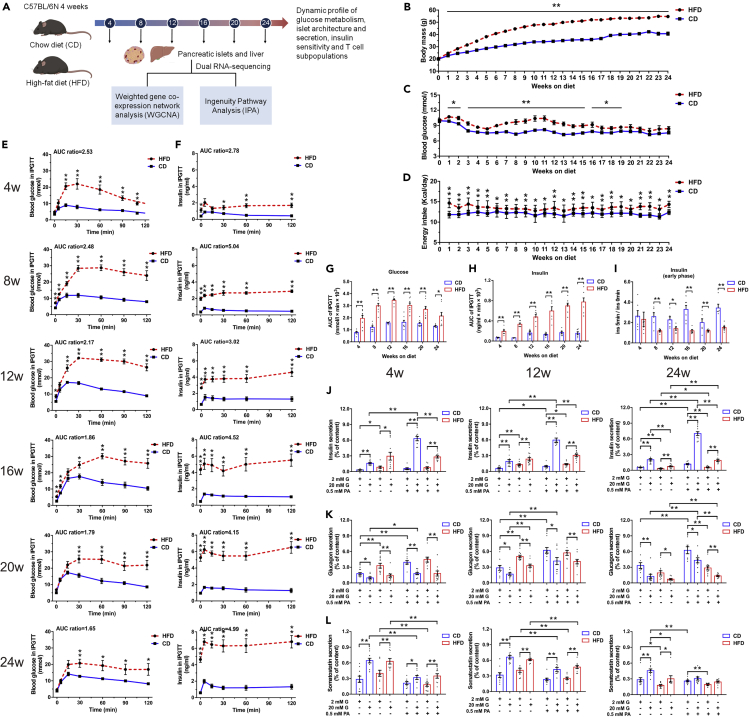
Progressively impaired glucose homeostasis and islet dysfunction of HFD mice both *in vivo* and *in vitro* (A) Schematic of the experimental design. (B–D) Weekly monitored body weight (N ≥ 16 mice/group) (B), morning ad libitum blood glucose (N ≥ 14 mice/group) (C), and caloric intake (N ≥ 4 cages/group) (D). (E and F) Blood glucose (E) and the corresponding plasma insulin concentration (F) during IPGTT (2 g/kg glucose) (N ≥ 6 mice/group). The ratios of AUC for HFD versus CD are presented at the top of each graph. (G and H) AUC calculation for glucose (G) and insulin (H) during IPGTT. (I) First-phase insulin secretion defect in HFD mice indicated by fold change of insulin level at 5 min with respect to 0 min. (J–L) Insulin (J), glucagon (K), and somatostatin (L) secretion in islets isolated from HFD and CD mice after 4, 12, and 24 weeks of diet (N = 4–5 mice/group). All data are expressed as mean ± standard error of mean (SEM) and analyzed using unpaired two-tailed t test. ∗p < 0.05, ∗∗p < 0.01. See also Figure S1.

To illustrate the global characteristics and molecular dynamics in HFD model, we monitored the temporal profile of glucose metabolism, islet architecture and secretion, and tissue-specific insulin sensitivity in C57BL/6N mice fed on a 60% HFD or a chow diet (CD) for 24 weeks. Transcriptomes of islets and liver at six consecutive time points with the interval of 4 weeks (week 4, 8, 12, 16, 20, and 24) were analyzed using weighted gene co-expression network analyses (WGCNA) and Ingenuity Pathway Analysis (IPA) (Figure 1A). As to non-fasting morning blood glucose level, we identified mild hyperglycemia after 1 week of HFD. From week 8 to week 14, largest difference in non-fasting plasma glucose was observed. Notably after week 20, although insignificant, plasma glucose in HFD remained ∼1 mmol/L higher than that of CD (Figure 1C), which is in accordance with findings previously described by Winzell et al. (Winzell and Ahren, 2004). There was also a modest increase in energy consumption in HFD mice compared with CD ones during the study period (Figure 1D). As expected, caloric excess was disproportionately stored in adipose tissue throughout 24 weeks in HFD group (Figure S1A). The biochemical measurements in Figure S1B presented HFD-induced hepatoxicity as revealed by increases in serum liver enzymes and aminotransferases and marked alternations of total cholesterol (T-CHO), low-density lipoprotein cholesterol (LDL-C), and high-density lipoprotein cholesterol (HDL-C). In concordance with impaired liver function, we also identified gradually deteriorated hepatic steatosis, cytoplasmatic ballooning, fibrosis, and angiogenesis in HFD mice (Figure S2A).

### Progressively impaired glucose homeostasis and islet function of HFD mice both *in vivo* and *in vitro*

Intraperitoneal glucose tolerance tests (IPGTTs) were conducted every 4 weeks to monitor islet function during 24 weeks of diet treatments. Compared with CD mice, HFD mice developed glucose intolerance as early as 4 weeks, presenting with a significantly higher peak and slower decay of plasma glucose following glucose challenge (Figure 1E). As a result, the integrated glucose level (area under the curve (AUC)) in HFD was around 2-fold of that seen in CD for the entire duration of 24 weeks (Figure 1G). However, this glucose intolerance slightly ameliorated after 16 weeks of HFD and fasting hyperglycemia attenuated at week 20, which may be attributable to the compensatory increase in β-cell function and/or mass in response to the diet. Indeed, the corresponding insulin level revealed a progressively exaggerated and significantly delayed glucose-induced secretory response in HFD mice (Figure 1F). The integrated insulin secretion (AUC) during glucose challenge increased from 2.78-fold (week 4) to 4.99-fold (week 24) of that seen in CD group (Figure 1H). Notably, we observed an evidently elevated fasting insulin level in HFD mice at week 4, prior to the development of fasting hyperglycemia at week 8. Despite the overall enhanced insulin secretion, the first-phase glucose-induced insulin secretion (insulin level at 5 min relative to basal release) was impaired from week 8 on HFD (Figure 1I). Fasted and 2 h refed plasma glucagon levels suggested α-cell dysfunction, and subsequently, inappropriate glucagon release was not responsible for hyperglycemia in HFD mice (Figure S1C).

We also measured insulin, glucagon, and somatostatin secretion in intact islets from mice on HFD or CD at week 4, 12 (with the highest blood glucose level during IPGTT), and 24 (with the highest serum insulin level during IPGTT) in response to low and high glucose in the presence or absence of palmitate (PA). The fold increase in insulin secretion stimulated by 20 mM glucose alone (3.53-fold in HFD versus 4.77-fold in CD at week 4; 1.81-fold in HFD versus 3.25-fold in CD at week 12; and 2.29-fold in HFD versus 3.62-fold in CD at week 24) or 0.5 mM PA alone (0.90-fold in HFD versus 1.72-fold in CD at week 4; 1.07-fold in HFD versus 1.65-fold in CD at week 12; and 1.79-fold in HFD versus 2.12-fold in CD at week 24) was both modestly reduced. In agreement with the previous study (Peyot et al., 2010), PA potentiated glucose-stimulated insulin secretion (GSIS) was also impaired at these three time points (3.72-fold in HFD versus 11.03-fold in CD at week 4, 2.20-fold in HFD versus 6.12-fold in CD at week 12, and 3.34-fold in HFD versus 5.95-fold in CD at week 24). It is noticeable that islets from 4 weeks to 12 weeks of HFD exhibited a higher basal insulin release (2 mM glucose in the absence of PA) in comparison with CD. However, after 24 weeks of treatment, insulin secretion of HFD mouse islets was significantly lower than CD with or without the presence of stimulatory glucose or PA concentration, indicating β-cell function failure (Figure 1J). Glucagon secretion of both HFD and CD mouse islets showed intact secretory response to glucose and PA at all three time points. Similar to that of insulin, a more robust basal release of glucagon was observed in HFD at week 4 and 12, albeit with an evident reduction at week 24 (Figure 1K). Somatostatin secretion in HFD mice displayed marginally decreased sensitivity in response to the stimulatory action of 20 mM glucose and the inhibitory action of PA at 20 mM glucose compared with CD. Similar to insulin and glucagon secretion, an elevated basal somatostatin release was only noticed in HFD islets at week 4 and 12, followed by a significantly reduced secretion at week 24 (Figure 1L). In addition, the pancreatic content of insulin was significantly higher in HFD mice at all three time points of feeding, whereas a slight increase in glucagon content was only observed at week 24, and no changes were identified as to somatostatin content (Figure S1D). Altogether, these results demonstrated specific hyposensitivity of HFD islets to glucose and PA despite enhanced basal insulin, glucagon, and somatostatin secretion at early stage.

### Dynamic changes in islet morphology, cell composition, and ultrastructure

**Figure 2:**
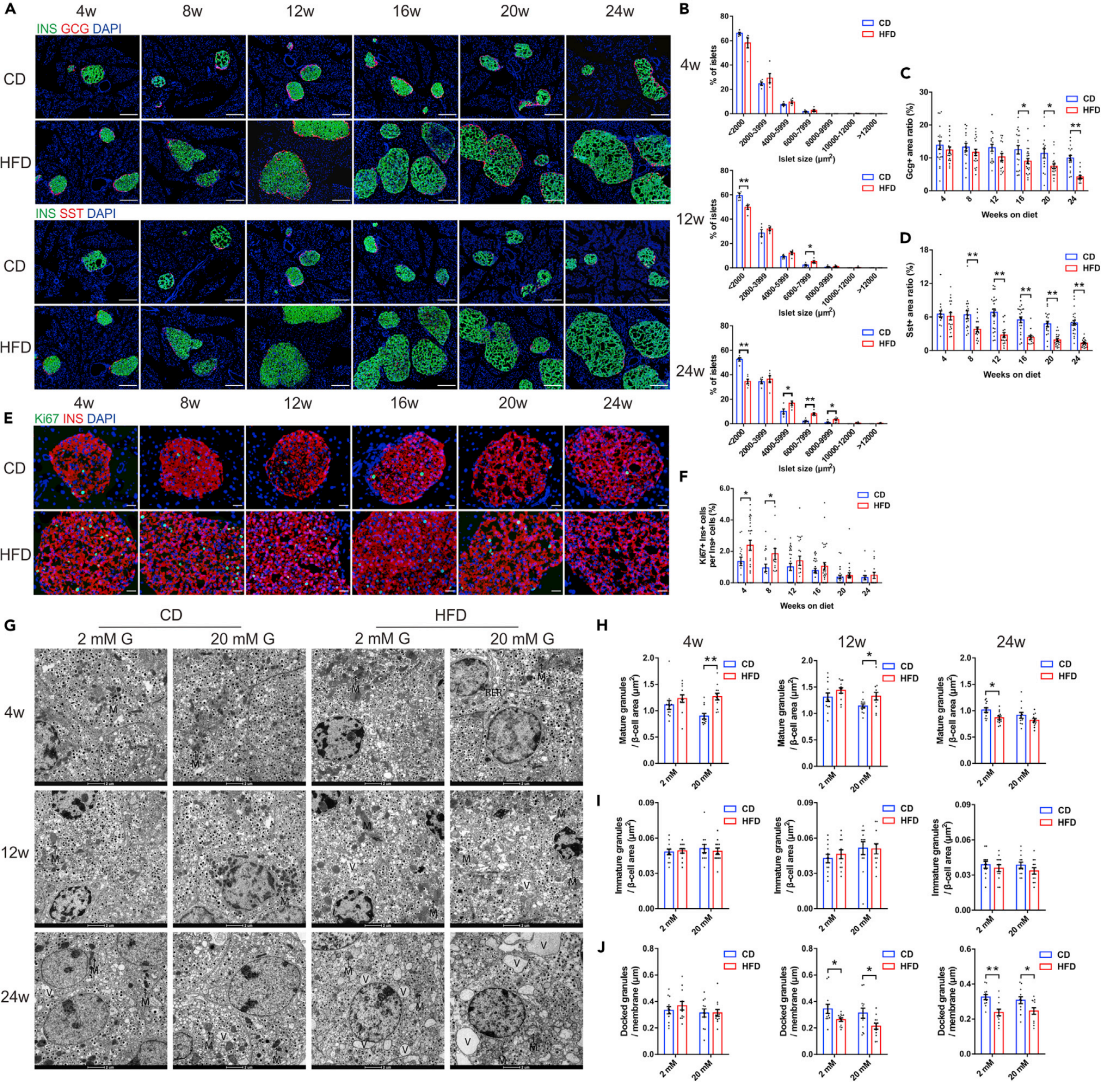
Dynamic changes in islet morphology, cell composition, and ultrastructure (A) Representative immunofluorescent images showing consecutive pancreatic sections double-labeled either for insulin and glucagon or insulin and somatostatin (scale bar: 50 μm). (B) Islet size distributions analyzed by morphometry (N = 5 mice/group). (C and D) Quantification of glucagon (C) and somatostatin (D) stained area (N = 15–25 islets/group, N = 4–5 mice/group). (E) Representative immunofluorescent images showing Ki67^+^ Ins^+^ cells in pancreatic sections (scale bar: 20 μm) (F) Quantification of the percentage of Ki67^+^ Ins^+^ cells in total Ins^+^ cells (N ≥ 14 islets/group, N = 3–5 mice/group). (G) Representative electron micrographs showing β-cells with insulin-containing granules after 2 mM or 20 mM glucose stimulation. Mitochondria (M), endoplasmic reticulum(RER), and vacuoles (V) are marked (scale bar: 2 μm). (H–J) Quantification of mature (H), immature (I), and docked (J) insulin granules in β-cells of HFD and CD islets (N = 12 β-cells/group, N = 6–8 islets/group, N = 3 mice/group). All data are expressed as mean ± SEM and analyzed using unpaired two-tailed t test. ∗p < 0.05, ∗∗p < 0.01. See also Figure S2.

Immunofluorescent staining was performed to examine the changes in islet size distribution and cell composition at different time points of diet treatments. In comparison with CD mice, the islet size did not change significantly after HFD feeding for 4 weeks, whereas at week 12, the prevalence of large islet population (6000–7999 μm^2^) doubled and that of small islets (<2000 μm^2^) relatively decreased in HFD group. The difference was even more profound after 24 weeks of HFD (Figure 2B). The glucagon positive area ratio of HFD islets began to decline from week 16 despite of concomitant augmentation in islet size (Figure 2C). The somatostatin positive area ratio of HFD islets also decreased by nearly 2-fold at week 8 and continued to reduce over 24 weeks of feeding period (Figure 2D). Pancreatic immunohistochemical examination corroborated the findings of progressively enlarged islet mass and increased abundance of Ins^+^ β-cells in HFD mice (Figure S2B). To test whether the hyperplasia of β-cells was due to increased proliferation, we next quantitated proliferation markers such as Ki67 in β-cells at different time points of diet treatments. In CD mice, Ki67^+^ Ins^+^ β-cells were barely detectable. The percentage of Ki67^+^ Ins^+^ β-cells was only transiently increased in HFD mice at week 4 and 8 and showed no significant difference to CD during the subsequent weeks (Figure 2F).

We next analyzed the ultrastructure of β-cells from HFD and CD mice by transmission electron microscopy (TEM). At week 4 and 12 of the diet, there was no significant difference in density of mature granules after 2 mM glucose treatment between two groups. However, 20 mM glucose stimulation led to an evident increase in mature granule density in HFD. Interestingly, in β-cells of mice fed on HFD for 24 weeks, the density of mature granules in 2 mM glucose was significantly reduced compared with CD. This supported the view from *in vitro* GSIS results that there was a transition from enhanced to impaired insulin secretion in HFD mice (Figure 2H). With regard to immature granules, no significant difference was observed (Figure 2I). We also calculated the density of docked granules and identified that HFD resulted in a different distribution of granules with fewer granules docked at the cell membrane compared with CD (Figure 2J). This may explain the reduced first phase of glucose-induced insulin secretion in HFD mice.

As previously reported (Gupta et al., 2017), β-cells from HFD mice also showed several ultrastructural alterations (Figure 2G). HFD β-cell mitochondria were round-shaped rather than elongated, with fragmented cristae, reduced electron density, and augmented volume. There was also massive accumulation of vacuoles characterized by the presence of closed membranes surrounding organelles and cytoplasmic portions in β-cells, possibly suggesting dysregulated autophagy. Interestingly, α-cells appeared ultrastructurally normal and well granulated, whereas δ-cells were characterized by degranulation in HFD-treated group (Figure S2C), which was quite similar with the TEM features observed in T2DM patients (Folli et al., 2018).

### Longitudinal assessment of systemic and tissue-specific insulin sensitivity in HFD mice

**Figure 3:**
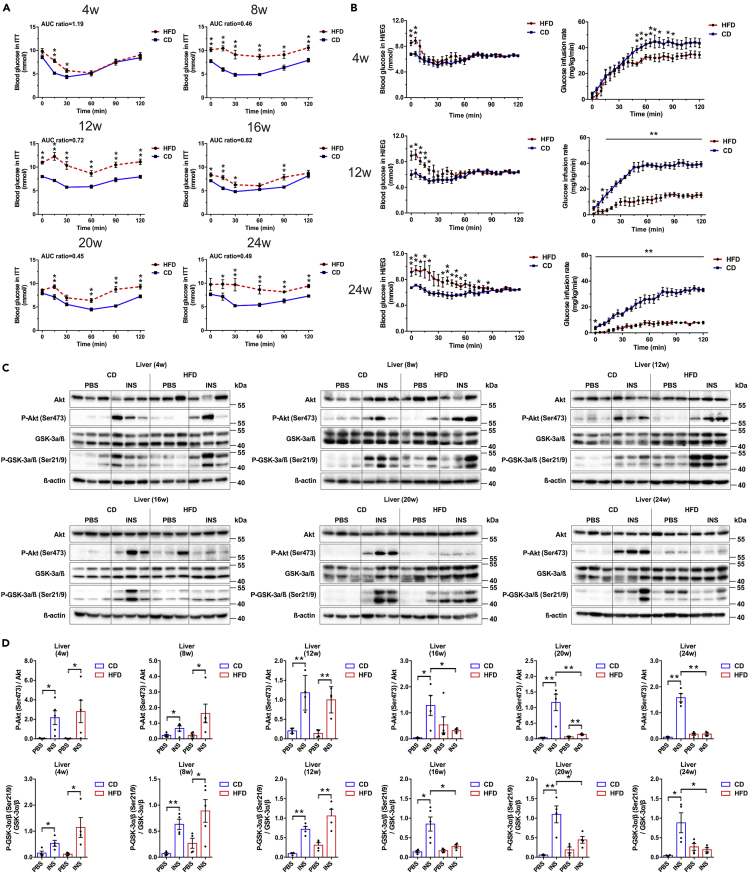
Longitudinal assessment of systemic and tissue-specific insulin sensitivity in CD and HFD mice (A) Blood glucose during ITT (0.75 U/kg insulin) (N ≥ 7 mice/group). The ratios of AUC for HFD versus CD are presented at the top of each graph. (B) Hyperinsulinemic-euglycemic clamp tests (N ≥ 6 mice/group). (C and D) Immunoblot (C) and quantification (D) of Akt (Ser473) phosphorylation status relative to total Akt and GSK-3α/β (Ser21/9) phosphorylation status relative to total GSK-3α/β in liver (N = 4–6 mice/group). All data are expressed as mean ± SEM and analyzed using unpaired two-tailed t test. ∗p < 0.05, ∗∗p < 0.01. See also Figure S3.

To assess whether the impaired glucose tolerance in HFD was due to defects in insulin sensitivity, longitudinal insulin tolerance tests (ITTs) were performed with an interval of 4 weeks. Compared with CD mice, HFD mice presented a relatively intact insulin-induced hypoglycemic response at week 4, whereas overt insulin resistance was only detectable after 8 weeks of diet treatment (Figure 3A). Hyperinsulinemic-euglycemic clamps (HI/EG) were also conducted to evaluate systemic insulin sensitivity. At week 4, HFD mice had a non-evident decrease in glucose infusion rate (GIR) compared with CD (insulin sensitivity index (ISI) = 0.90 ± 0.08 in HFD versus ISI = 1.17 ± 0.10 in CD at week 4, p = 0.060). However, after 12 and 24 weeks of treatment, a significant lower GIR was required for HFD mice to maintain euglycemia at steady state (ISI = 0.42 ± 0.14 in HFD versus ISI = 1.79 ± 0.43 in CD at week 12, p = 0.013; ISI = 0.15 ± 0.04 in HFD versus ISI = 0.98 ± 0.16 in CD at week 24, p < 0.001) (Figure 3B).

For tissue-specific insulin action as displayed in Figures 3C and 3D, the hepatic acute response to insulin was not different between HFD and CD before week 16. However, phosphorylation of Akt after insulin injection was significantly lowered by 75.46% (week 16), 87.85% (week 20), and 88.34% (week 24) afterward. The phosphorylation of GSK-3α/β in liver also demonstrated similar patterns. As regard to quadriceps femoris (Figures S3A and S3B) and gastrocnemius tissue (Figures S3C and S3D), the insulin-stimulated increases in p-Akt and p-GSK-3α/β were also blunted in HFD starting from week 16. Of note, in adipose tissue (Figures S3E and S3F), reduced insulin response was evident since week 8 on HFD, prior to that in liver and skeletal muscle. This phenomenon might be attributed to adipose tissue being the primary site of nutrient storage. These data revealed that the development of insulin resistance at different organs had variable time course, with liver and skeletal muscle initiating from week 16 and adipose tissue starting from week 8.

### Transcriptomic profiles and pathway dynamics of islets and liver during diabetes progression

**Figure 4:**
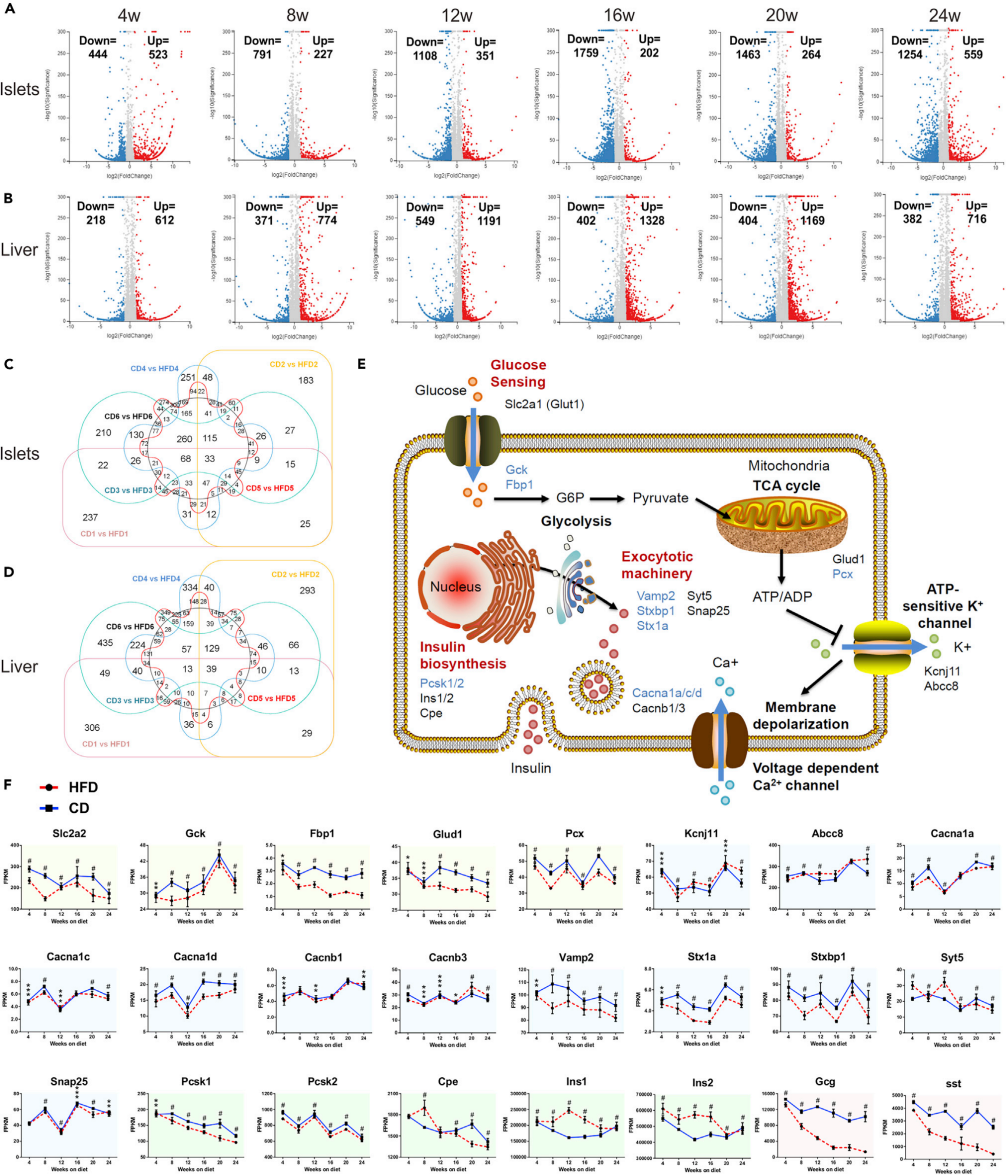
Transcriptomic profiles and pathway dynamics of islets and liver during diabetes progression (A and B) Islet (A) and liver (B) volcano plots. The numbers of upregulated (red dots) and downregulated (blue dots) genes are marked in each graph. (C and D) Islet (C) and liver (D) Venn diagrams of identifiable and quantifiable DEGs. (E) Schematic representation illustrating the physiology and key genes of GSIS in pancreatic β-cells. Genes that were significantly downregulated are colored in blue. (F) The time course expression data for genes involved in GSIS. Yellow, blue, green, and red shaded areas, respectively refer to genes participating in glucose sensing, exocytotic machinery, insulin biosynthesis, and the secretion of other islet cells. All data are expressed as mean ± SEM and analyzed using unpaired two-tailed t test. ∗adjusted p < 0.05, ∗∗adjusted p < 0.01, ∗∗∗adjusted p < 0.001, ^#^adjusted p < 0.0001. See also Figures S4–S6.

To unveil the molecular mechanisms underlying these metabolic changes during diabetes progression, RNA sequencing and transcriptomic analyses of islets and liver were respectively performed in quadruplicates at six consecutive time points of diet treatments (week 4, 8, 12, 16, 20, and 24). In total, 3,844 differentially expressed genes (DEGs) were found in islets, of which 33 were shared among all six time points (Figure 4C). With regard to liver, 4,101 DEGs were discovered throughout 24 weeks of feeding, of which 39 were overlapped (Figure 4D). To validate the transcriptomic results, 10 DEGs were randomly selected, and their expression patterns measured by quantitative real-time PCR (qRT-PCR) in independent HFD/CD islet and liver samples were very similar to those in RNA-sequencing data (Figures S4A and S4B).

For pathway dynamics analyses, we investigated the temporal patterns with representative genes involved in islet hormone secretion, pancreatic endocrine cell development, and hepatic insulin resistance. Figures 4E and 4F depicted the primary signal flow of GSIS in pancreatic β-cells, showing some downregulated genes in glucose sensing and metabolism (*Gck and Fbp1*), Ca^2+^ flux (*Cacna1d*), granule docking, and release (*Vamp2*, *Stxbp1 and Stx1a*). The pathway of insulin biosynthesis (*Cpe* and *Ins1*) was upregulated before week 16, whereas gradually downregulated afterward, which might indicate a transition from compensatory oversecretion to functional impairment. We also concluded the gene expression of a synopsis of factors regulating endocrine pancreas development over time (Figure S5). Certain genes participating in the differentiation of pancreatic progenitors into endocrine progenitors (*Gata6*, *Sox9* and *Onecut1*) were downregulated throughout 24 weeks. Furthermore, Figure S6 provided a schematic representation of mechanisms involved in hepatic insulin resistance. Pathways of lipid-induced insulin resistance (PKCε (*Prkce*) and PP2A (*Sptlc1/2* and *Ppp2ca*)), intracellular inflammatory signaling (TLR4 (*Tlr4*) and IKK (*Ikbkb*)), and unfolded protein response (IRE1α (*Ern1*), BiP (*Hspa5*) and PERK (*Eif2ak3*)) were significantly upregulated in HFD mice.

**Figure 5:**
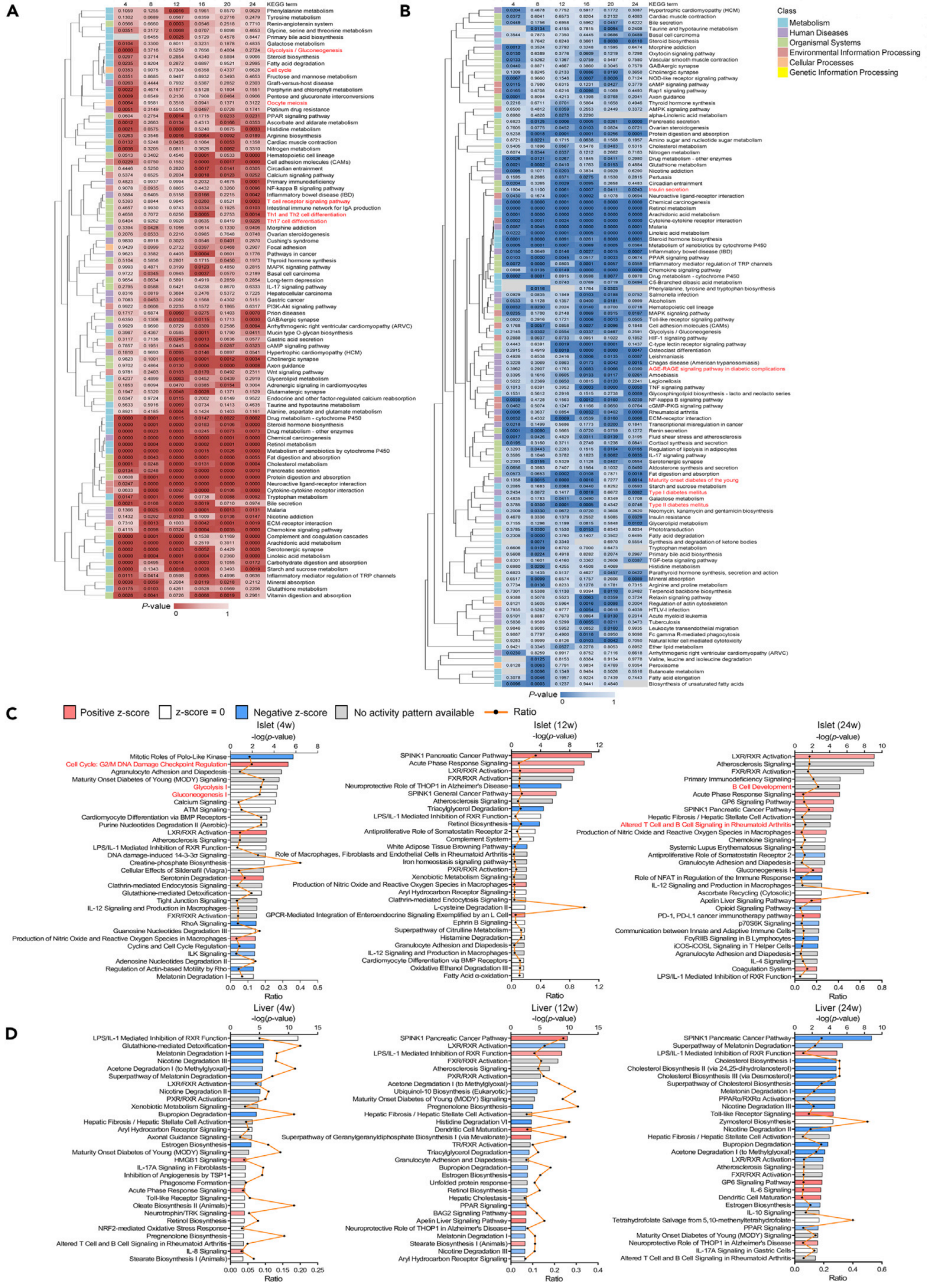
Temporal pathway enrichment analyses of DEGs (A and B) Clustered heatmaps of islets (A) and liver (B) showing KEGG pathway enrichment analyses of DEGs across six time points. The top 40 enriched KEGG pathways ordered in a decreasing level of significance (p value, Fisher's exact test) were first identified in islets and liver at each time point. We then took the union of six time points and clustered by columns using average linkage method. The intensity of color represents p value. (C and D) IPA of islets (C) and liver (D) presenting the top 30 canonical pathways (y axis) modulated by DEGs at week 4, 12, and 24 of diet. These IPA pathways were sorted by the significance of association between dataset and canonical pathway (log of p value, Fisher's exact test, top x axis). The ratios of genes that map to each canonical pathway (bottom x axis) were also presented. See also Figure S7 and Table S1.

Kyoto Encyclopedia of Genes and Genomes (KEGG) enrichment (Figures 5A and 5B) and IPA (Figures 5C and 5D) were carried out in each tissue to demonstrate top overrepresented canonical pathways sorted by p value annotating for DEGs. In islets, the early perturbations were characterized by upregulation of cell replication pathways (cell cycle and oocyte meiosis in KEGG; cell cycle: G2/M DNA damage checkpoint regulation in IPA; marked in red), which was coincident with the assessment of proliferative antigens in histological staining. Another notable feature is the gradual upregulation in glycolysis/gluconeogenesis (marked in red) since week 4 on HFD, probably suggesting that defect in oxidative phosphorylation would lead to the anaerobic metabolism being dominant in producing energy. During week 16–24, signaling pathways associated with adaptive immune responses were significantly enriched in islets (T cell receptor signaling pathway, Th1 and Th2 cell differentiation, and Th17 cell differentiation in KEGG; B cell development, and altered T cell and B cell signaling in rheumatoid arthritis in IPA; marked in red and listed in Table S1). It is also noticeable that some enriched pathways in liver were correlated with islet function and diabetic progression, such as insulin secretion, type I and type II diabetes, maturity onset diabetes of the young, and AGE-RAGE signaling pathway in diabetic complications (marked in red).

### Weight gene co-expression network analyses presenting trait-correlated modules

WGCNA algorithm was used in islets and liver to define trends in gene co-expression and look for a consensus network of genes that were correlated in all conditions. In total, we detected 42 co-expression modules in islets and 65 co-expression modules in liver (Table S2). To identify gene clusters associated with traits of islet and liver function together with other metabolic characteristics (weight, energy intake, fasting glucose, fasting insulin, and other derivative parameters from IPGTT and ITT, alanine aminotransferase (ALT), aspartate aminotransferase (AST), alkaline phosphatase (ALP), albumin (ALB), T-CHO, HDL-C, LDL-C, and triglyceride (TG), listed in Table S3), we used linear regression models treating the module eigengenes (MEs) as dependent variables and the traits as independent variables. The individual metabolic profile was retrieved from the same mice subjected to RNA sequencing. Those module-traits relationships were further used to select biologically meaningful modules for downstream analyses.

In total, eight islet modules were found to have a significant correlation with any one of the insulin secretion parameters calculated from IPGTT experiments. Genes from these modules were pooled and further analyzed by KEGG as shown in the top 30 annotated pathways. We observed enrichment of proliferative genes (*Cdc20*, *Bub1*, *Ccna2*, *Ccnb2*, and *Cdk1*) in salmon module and energy-metabolism-related genes (*Atp5c1*, *Ndufs4*, *Cox5b*, *Cox7b*, and *Ppa2*) in lightcyan1 and darkgrey modules (Figure S7A). Notably, patterns of liver module expression across various traits exhibited strong and negative correlation with islet function, which might be attributed to liver as a crucial organ regulating systemic metabolism and glucose homeostasis. In liver, modules of interest were defined as those significantly correlated with more than five insulin secretion parameters. Among the 26 modules of interest, KEGG pathway enrichment suggested that brown and blue modules were evidently related to immune and inflammatory responses, including TNF signaling pathway (*Junb*, *Pik3cd* and *Ccdc88b*), toll-like receptor signaling pathway (*Tlr6*, *Ccl3* and *Ticam2*), T cell receptor signaling pathway, and B cell receptor signaling pathway (*Jun*, *Fos*, *Cd22*, *Blnk* and *Vav1*) (Figure S7B).

### Proliferative and immune response signaling pathways underlying the crosstalk between islets and liver

**Figure 6:**
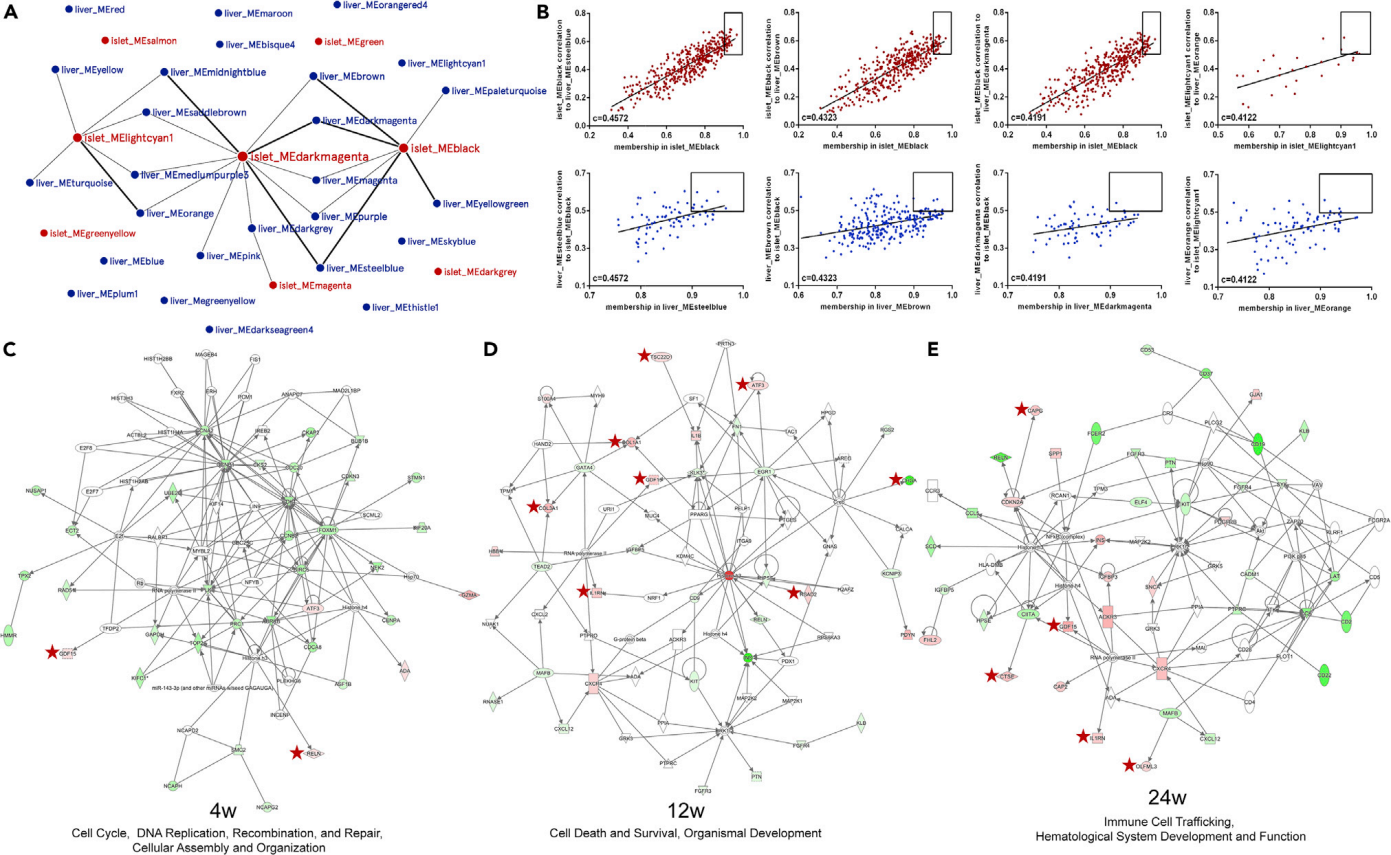
Key genes and signaling pathways involved in the crosstalk between islets and liver (A) Network of trait-correlated islet and liver modules. In total, 8 islet (red circles) and 26 liver (blue circles) modules were defined as associated with insulin secretion traits. Among these 208 (8 × 26) pairs, 8 pairs of highly correlated modules were identified with Pearson coefficient >0.4 (linked with thick lines) and 17 pairs showed coefficient >0.35 and ≤0.4 (linked with thin lines). (B) Examples of key gene identification between two highly related trait-correlated islet and liver modules (Pearson coefficient >0.4). Each dot represents one gene and “key genes” (the top right of each graph) are those most correlated to a counterpart partner module (correlation >0.5) and also displayed a high module connectivity (membership >0.9). (C–E) Ingenuity network analyses using islet DEGs as the basis and sequentially adding liver DEGs encoded for proteins that can be secreted at the same time point. The most dominant networks of week 4 (C), 12 (D), and 24 (E) were depicted. The upregulated genes are in red and the downregulated are in green. The molecules marked with stars are derived from liver, and the rest are derived from islets. See also Figures S4 and S7, and Table S2, S3, S4 and S5.

Following the single-tissue, single-platform analyses of individual dataset, we considered the inter-tissue, multi-platform analyses using WGCNA and IPA by combining the islet and liver transcriptomic data together. To evaluate co-ordinated molecular interactions by identifying genes of islets and liver that were highly correlated, we generated a massive matrix of cross-tissue Pearson correlation coefficients for each gene of the islet modules to each gene of the liver modules in WGCNA. By taking the gene connectivity (membership) within its own module into consideration, combined correlation coefficients for each pair of islet and liver modules were calculated. In order to search for potential molecules underlying the inter-tissue communication contributing to islet dysfunction, we further looked into pairs of biologically meaningful modules, including 8 islets and 26 liver modules that were in association with insulin secretion traits. Among 208 (8 × 26) pairs, 8 pairs were identified with Pearson coefficient >0.4 and 17 pairs showed coefficient >0.35 and ≤0.4 (Figure 6A). We also detected “key genes” within a given module by calculating the gene's correlation to a partner module. Within 8 pairs of islet and liver modules (Pearson coefficient >0.4), 34 islet and 25 liver “key genes” (Table S4) in total that were highly representative of their own modules (membership >0.9) and highly correlated to a counterpart partner module (correlation>0.5) were found (examples of correlation between 4 pairs of modules as shown in Figure 6B). In islets, 23 “key genes” were differentially expressed between HFD and CD, which included genes encoding immune-related molecules (*Cxcr4* and *Fosb*) and transcription factors (*Egr1*). Early growth response 1 (EGR-1) was found to attenuate palmitic-acid-induced ER stress and apoptosis in β-cells (Cheong et al., 2015). Among 17 differentially expressed “key genes” in liver, it is noticeable that C-X3-C motif chemokine receptor 1 (CX3CR1) is upregulated. Elevation of CXCR1 expression was also reported in liver following ischemia-reperfusion injury, mediating hepatic inflammation (Clarke et al., 2011). Interestingly, a recent study has demonstrated that C-X3-C motif chemokine ligand 1 (CXCL1), the specific ligand for CXCR1, can be produced and secreted from human islets, most likely from α-cells (Rutti et al., 2014). These might suggest a possible role of CX3CL1-CX3CR1 in liver-islet crosstalk.

To relate islet transcriptomic changes with liver perturbations, IPA was also applied for generating networks and identifying major integrative hubs. We postulated that one of the mechanisms underlying the islets-liver crosstalk might entail the direct effect of liver-derived secretome on islets. Consequently, we constructed networks for each time point by using the islet DEGs as the basis and sequentially adding the liver DEGs encoded for proteins that can be secreted. Top scored networks showed potential interactions between several genes and secreted molecules implicated in cell cycle, DNA replication, recombination and repair during week 4 (Figure 6C, liver-derived molecules marked with stars), cell death and survival, organismal development around week 12 (Figure 6D, liver-derived molecules marked with stars), and immune cell trafficking, hematological system development and function at week 24 (Figure 6E, liver-derived molecules marked with stars) (Table S5). Embedding the liver dataset allowed the identification of several interconnected molecules, including growth differentiation factor 15 (GDF15, in top scored network of week 4, 12, and 24), secreted protein acidic and rich in cysteine (SPARC, in second top scored network of week 4, data not shown), and activating transcription factor 3 (ATF3, in top scored network of week 12) as possible liver-derived candidates correlated to alternations in islet transcriptomics. The upregulations of GDF15 and SPARC in liver of HFD mice are in accordance with the previous findings (Mazzolini et al., 2018; Patel et al., 2019), and the increased expressions of ATF3 at all three time points were further verified by western blotting (Figures S4C and S4D).

### The imbalance of T cell subpopulations in the development of diet-induced diabetes

**Figure 7:**
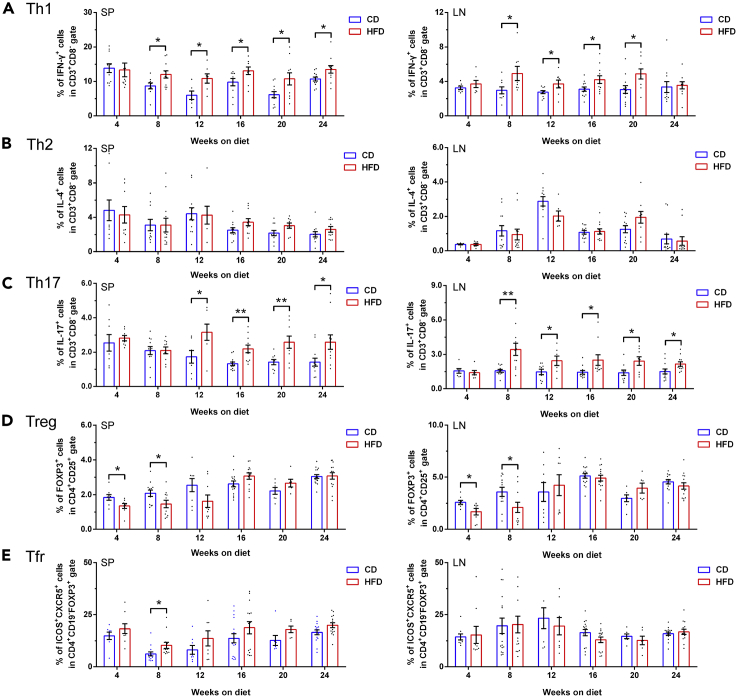
Characterization of splenic and lymphonodular T cell subpopulations during 24 weeks of feeding (A–E) The frequencies of splenic (left column of scatter dot plots) and lymphonodular (right column of scatter dot plots) CD3^+^CD8^−^IFN-γ^+^ Th1 cells (A), CD3^+^CD8^−^IL-4^+^ Th2 cells (B), CD3^+^CD8^−^IL-17^+^ Th17 cells (C), CD4^+^CD25^+^FOXP3^+^ Tregs (D), and CD4^+^CD19^−^FOXP3^+^ICOS^+^CXCR5^+^ Tfr cells (E) (N ≥ 7 mice/group). All data are expressed as mean ± SEM and analyzed using unpaired two-tailed t test. ∗p < 0.05, ∗∗p < 0.01. See also Figure S4.

The onset or progression of obesity-related diabetes can be triggered by chronic inflammation, which is speculated to be induced by high levels of fatty acids persistently activating immune system (Gregor and Hotamisligil, 2011; Odegaard and Chawla, 2013). Our WGCNA and IPA both suggested that T-cell-mediated immune responses are involved in the development of HFD-induced diabetes and underlying mechanisms of the islet-liver crosstalk. To further validate this postulation, we used flow cytometry to verify the temporal frequencies of T cell subpopulations both in spleens and draining lymph nodes during 24 weeks of feeding (Figure S4E). The proportion of splenic and lymphonodular T helper 1 (Th1) cells were markedly increased in HFD mice compared with control mice since week 8 (Figure 7A). The disequilibrium of T helper 17 (Th17) cells also exhibited similar tendency as Th1 cells with elevated frequencies (Figure 7B), whereas the percentage of T helper 2 (Th2) cells were comparable between two groups (Figure 7C). Interestingly, a significant reduction of regulatory T cells (Tregs) during the early stage of obesity progression was also found (Figure 7D), whereas no difference was detected with regard to T follicular regulatory (Tfr) cells (Figure 7E). Alternations of T cell compartment exhibited a priming stage of decrement in anti-inflammatory Tregs at week 4–8 and an amplification stage of increase in proinflammatory Th1 and Th17 cells at week 12–24. However, as shown in Figure S2D, no prominent insulitis or infiltration of CD3^+^ lymphocytes was present in HFD islets throughout the 24 weeks of diet treatment.

## Discussions

Despite decades of research, the absolute timeline and primary driving event after initiation of nutritional excess are yet to be determined in diabetic rodent models. The inconsistency can be attributed to confounding factors including genetic differences among strains, sex, age of diet onset and its duration, and diet composition. In the current study, we sought to avoid these variables by using the C57BL/6N mice with extended span. C57BL/6N mice do not harbor the mutation of nicotinamide nucleotide transhydrogenase (NNT) ,which has been associated with impaired β-cell function and glucose intolerance in some studies (Aston-Mourney et al., 2007; Freeman et al., 2006; Toye et al., 2005). Although some of the changes in glucose homeostasis we described here are well in agreement with what has been well acknowledged regarding HFD mouse model, including gradually developed glucose intolerance (Winzell and Ahren, 2004), hyperinsulinemia, insulin resistance, impaired insulin secretion to PA (Peyot et al., 2010), β-cell compensation by proliferation at early stage (Roat et al., 2014; Sone and Kagawa, 2005), and changes in β-cell morphology by TEM (Gupta et al., 2017), the novelty of our study lies in (1) systematically describing the metabolic characteristics over a long period of observation to define the onset of metabolic defects during HFD; (2) combining phenotyping together with temporal transcriptomic screening to highlight the gradual molecular and functional changes in key glucose-regulating organs; and (3) involving two critical tissues in glucose metabolism, islets (responsible for hyperinsulinemia) and liver (involved in insulin resistance), to identify potential interconnected genes participating in this inter-tissue crosstalk.

We identified that mild fed hyperglycemia was maintained in HFD mice throughout 24 weeks of diet and gradually alleviated by an ordered succession of adaptive β-cell response. Around week 4, fasting hyperinsulinemia first occurred in the absence of detectable fasting glucose elevation and impaired systemic insulin sensitivity. A plausible explanation for the glucose intolerance observed at week 4 despite of hyperinsulinemia and relatively intact insulin sensitivity is probably due to disturbed pulsatile pattern of insulin secretion in HFD mice. Changes in β-cell pulsatility could impact the efficacy of secreted insulin on its targets, particularly suppressing hepatic glucose production (Matveyenko et al., 2012; Meier et al., 2005) and enhancing uptakes by peripheral tissues (Satin et al., 2015). These impacts, however, could not be detected by HI/EG clamps with constant infusion of insulin and western blot of targeted tissues following an intraperitoneal insulin injection at week 4. From week 4–16, HFD mice exhibited progressively exaggerated insulin secretion in tandem with evident hyperglycemia. Meanwhile, defective tissue-specific insulin responsiveness showed variable time course with liver and skeletal muscle developing insulin resistance from week 16, whereas adipose tissue starting from week 8. After week 12, we observed robust immune and inflammatory response both in spleen and draining lymph nodes, which could further aggravate systemic insulin resistance. Interestingly, glucose intolerance was slightly improved as indicated by glycemic levels during IPGTT conducted around week 16–24. This phenomenon might be due to a compensatory increase in β-cell mass, and the subsequent hyperinsulinemia can fully compensate or even overtake the adverse effect of insulin resistance in maintaining glucose homeostasis. A major technical problem in assessing the roles of hyperinsulinemia and insulin resistance in established obesity is that the measurement of each parameter may not be sufficiently precise and sensitive to dissect a chronological or causal sequence. Another potential issue of p-Akt and p-GSK-3α/β measuring is that insulin resistance in HFD-fed rodents could be possibly reversed by an overnight fast, which is a necessary procedure before administering a bolus of insulin (Han et al., 2009). Therefore, it would be premature to conclude that primary hyperinsulinemia initially causes insulin resistance.

Studies focusing on very early time points after the start of overfeeding could be more accurate and comprehensive to tease out the timeline. In rodent models, significant fasting hyperinsulinemia was presented around 3–4 days of HFD prior to hyperglycemia (Ben-Shlomo et al., 2012; Scherer et al., 2012; Turner et al., 2013; Waise et al., 2015) or insulin resistance (Gupta et al., 2017; Mosser et al., 2015). Some of these studies also revealed an early increase in body weight (Scherer et al., 2012; Waise et al., 2015), circulating free fatty acids (Scherer et al., 2012) and adipose tissue mass (Turner et al., 2013; Waise et al., 2015). However, systemic insulin resistance shown by a significant decrease of Akt phosphorylation in liver can only be detected after 14 days of HFD (Shiwa et al., 2015). Similar findings were also identified in human subjects that the first measurable change that occurs during overfeeding is usually an elevated fasting level of insulin concentration (Boden et al., 2015; Cahill et al., 2011; Lagerpusch et al., 2012; Wadden et al., 2012), not glucose. Nevertheless, it is still necessary to point out that both mechanisms (hyperinsulinemia leading to insulin resistance or insulin resistance triggering hyperinsulinemia) are not mutually exclusive and probably act in parallel at later stage.

Regardless of whether the trigger is overnutrition, insulin resistance, or both, the transition from adaptive β-cell response to pathological β-cell response represents a crucial step in the development of obesity-induced diabetes. We concluded the greatest enhancement in β-cell proliferation was observed at week 4 on HFD, whereas a continuous increase in β-cell area was only apparent until week 8–12. This seemingly unexpected delay also takes place in maternal islets during pregnancy (Butler et al., 2010), when highly productive β-cells have to divide and accumulate before identifiable enlargement. We also noticed, despite of the comparable proliferation rate between two diet regimens during week 12–24, a gradual growth in β-cell area was still detectable even at week 24, probably owing to the larger base of potentially proliferative β-cells in HFD mice. Combining our phenotypic characteristics together with transcriptomic datasets, we dissected the possible factors and pathways directly or indirectly involved in the β-cell stimulation facilitated by HFD. The nutrient metabolism in β-cells regulates not only insulin secretion but also cell proliferation (Moulle et al., 2017). We observed a significant upregulation of lipid-mediated proliferative signaling in islets as early as week 4 including G-protein-coupled receptor 119 (GPR119, receptor for 2-monoacylglycerol), CD36 (membrane transporter for fatty acids), long-chain acyl-CoA synthetase (ACSL), and heparin-binding epidermal growth factor (HB-EGF)-like growth factor (Zarrouki et al., 2014), which was in accordance with hyperlipidemia detected since week 4. For glucose-induced β-cell proliferation, carbohydrate response element-binding protein (ChREBP) (Metukuri et al., 2012; Zhang et al., 2015) was found upregulated after week 12. Insulin is known to modulate β-cell mass expansion directly via downstream signaling of insulin receptor (IR) and indirectly via the nuclear hormone receptor peroxisome proliferator-activated receptor γ (PPARγ) (Kulkarni et al., 2012; Linnemann et al., 2014). In agreement with hyperinsulinemia in HFD mice, elevated expressions of IR, insulin receptor substrate 2 (IRS-2), phosphoinositide 3-kinase (PI3K), phosphoinositide-dependent kinase-1 (PDK1), and PPARγ were identified across 24 weeks of feeding. In addition, as a strong candidate stimulating β-cell proliferation (Alvarez-Perez et al., 2014), HGF was noticed to be highly expressed in HFD liver, and its c-Met pathway was correspondingly upregulated in HFD islets. Our islet transcriptome also showed the perturbations of some other molecules associated with β-cell adaptive response, such as the upregulation of glucagon-like peptide-1 receptor (GLP-1R) (Buteau, 2008) and interleukin (IL)-6 receptor (Linnemann et al., 2017).

Despite of compensation illustrated earlier, T2DM is distinctively characterized by a progressive reduction in β-cell function preceding the onset of overt disease. Our secretion data *in vitro* displayed a hypothesized temporal transition of HFD islets at three stages. Initially, enhanced β-cell function compensated insulin resistance by higher insulin release without significant β-cell mass increase. During the second stage, β-cell hyposensitivity to glucose and PA started to appear alongside with elevated basal secretion. Adaptive β-cell response, in combination with mass expansion, contributed to the rising plasma insulin concentration. Subsequently at the third stage, insufficient β-cell function was shown by decreased basal release and defective response to stimuli despite the ongoing β-cell mass increment. This transition of β-cell dysfunction was also validated by alternations in β-cell calcium dynamics (Chen et al., 2016), as proposed by many other studies (reviewed in (Hudish et al., 2019)). However, we also note that these diet-induced mice models for diabetes cannot fully simulate human T2DM of which its pathogenesis is partially attributable to the progressive loss of β-cell mass (Butler et al., 2003; Kloppel et al., 1985; Rahier et al., 2008; Sakuraba et al., 2002; Yoon et al., 2003).

It has long been appreciated that activation of innate immune system contributes to β-cell dysfunction and destruction in T2DM, showing signs of sterile low-grade inflammation process in the pancreatic islets (Donath et al., 2013; Donath and Shoelson, 2011). A recent study unveiled that toll-like receptors TLR2 and TLR4 in islets may integrate inflammatory signals in diet-induced obesity to attenuate adaptive changes that govern β-cell replication (Ji et al., 2019). Our pathway enrichment analyses of islets not only demonstrated a tight interplay between glucose metabolism and innate immune response but also raised the intriguing possibility that T-cell-mediated immunity plays a role at later stage of HFD. As further validated by flow cytometry, alternations of T cell compartment exhibited a priming stage of decrement in anti-inflammatory Tregs at week 4–8 and an amplification stage of increase in proinflammatory Th1 and Th17 cells at week 12–24. However, these findings do not indicate the development of type 1 diabetes mellitus (T1DM), which results from T-cell-mediated autoimmune destruction of β-cells (Roep, 2003) in HFD model. A large amount of evidence has suggested a similar imbalance in T cell differentiation with a polarity shift toward Th1 and Th17 cells in obese T2DM patients (Sell et al., 2012; Touch et al., 2017; Zhou et al., 2018). It was also shown that the percentage of Tregs was decreased in peripheral blood of T2DM patients, especially in newly diagnosed diabetics (Jagannathan-Bogdan et al., 2011; Yuan et al., 2018).

Notably, significant insulitis or extensive infiltration of CD3^+^ T cells as previously described in HFD islets by Omar et al. (Omar et al., 2013) was not identified in our model. This inconsistency can be due to the differences in the age of diet onset and duration of diet treatment. Omar et al. observed clear pancreatic inflammation in 10-month-old mice fed chronically with HFD (duration of 11 months) (insulitis evaluation at 21 months old), which echoed the initial discovery by Hayashi et al. that autoimmune lesions of islets were evident in mice older than 12 months (Hayashi et al., 1989). Nevertheless, our results are in agreement with another study with shorter duration conducted earlier by the same group (Ahren et al., 2010), which Omar et al. described in the latter study that no similar inflammatory pathology was shown (Omar et al., 2013). Thus, it is possible that inflammation and lymphocytes infiltration in islets of HFD mice is age dependent and more likely to happen in older animals. Similarly, in human T2DM islets, number of CD3^+^ T cells was not significantly different from that in islets of non-diabetic donors (Butcher et al., 2014; Ehses et al., 2007). It remains unclear how adaptive immune response induced by nutritional stress is linked with T-cell-related genes in islets without apparent increase in T cell infiltration. Considering some cytokine receptors related to T-cell-mediated immunity are differentially expressed in HFD islets as shown in Table S1, it is possible that pro-inflammatory cytokines could mediate this process distally (Xia et al., 2017). Future studies focused on alternations of T cell subpopulation in peripancreatic and intrapancreatic lymph nodes could potentially provide the missing piece of this puzzle.

Liver is an ideal candidate for crosstalk with islet β-cells considering several reasons: (1) they both have a common embryonic origin; (2) shared portal system provides a transferring path for multiple metabolites and hormones regulating complex transcriptional networks; (3) liver plays a crucial role in glucose metabolism and the development of insulin resistance during T2DM (Tilg et al., 2017). In our study, inter-tissue, multi-platform analyses combining the islet and liver transcriptomes showed that potential interactions of genes were implicated in cell cycle during week 4, organismal development around week 12, and immune cell trafficking at week 24. Besides HGF and SerpinB1 that have been already reported, we also found several genes that are noteworthy. GDF15, as an important signal in response to nutrition stress induced by long-term high-fat feeding, has been identified recently to improve insulin sensitivity (Jung et al., 2018; Lee et al., 2017) and protect against cytokine-induced β-cell apoptosis (Nakayasu et al., 2020). We observed an upregulation of *Gdf15* in liver of HFD mice throughout 24 weeks of feeding and identified it in the top scored IPA networks of all time points as an interconnected gene with islets (Figures 6C–6E). Other potential molecules such as SPARC and ATF3 might also be involved in the inter-tissue crosstalk. SPARC is a secreted extracellular matrix protein expressed in many cell types including adipocytes (Chavey et al., 2006), hepatic cells (Mazzolini et al., 2018), and pancreatic cells (Ryall et al., 2014). SPARC secreted from adipose tissue could induce ectopic lipid deposition and insulin resistance (Kos and Wilding, 2010), whereas SPARC secreted from pancreatic stellate cells mediates the communication between stromal cells and endocrine cells by regulating β-cell survival (Ryall et al., 2014). SPARC has recently been identified to modulate β-cell glucose sensing through maintaining glucose transporter 2 expression level (Atorrasagasti et al., 2019) and promote insulin secretion via downregulation of RGS4 protein (Hu et al., 2020). ATF3 is involved in glucose metabolism in a variety of organs and tissues, whereas the physiological roles of ATF3 in the pancreas present crucial variances. Lee et al. concluded that pancreas- and hypothalamus-specific ATF3 knockout mice exhibit improved glucose tolerance (Lee et al., 2013). Zmuda et al. demonstrated that ATF3 plays a beneficial role by helping β-cells to cope with higher metabolic demand in the HFD-induced diabetes (Zmuda et al., 2010). However, the roles of these potential molecules in liver linking to islet function still remain elusive.

In conclusion, our study depicts a comprehensive landscape of temporal changes in islet and liver gene expression together with metabolic characteristic in HFD mice for 24 weeks. HFD mice exhibited progressively impaired glucose homeostasis with evident hyperinsulinemia and first-phase insulin secretion defect after week 4. The diverse secretory patterns of α-, β-, and δ-cell in response to glucose and PA indicated dynamic islet function deteriorating from dysregulation to failure. HFD islet morphology showed increased abundance of β-cells whose proliferation peaked at week 4, with concomitant reduction in δ-cell and α-cell proportion. Ultrastructure of β-cell also presented decreased docked granules with deranged cristae of mitochondria. We identified impaired systemic insulin sensitivity from week 12 with variable time course in tissue-specific insulin action. Overall, these phenotypic changes are in line with previous studies of high-fat fed C57BL/6 mice. Our islet and liver RNA-sequencing datasets outlined the impact of HFD on dynamics of molecular network at different stages. Correlation analyses of islet and liver modules with metabolic phenotypes illustrated that these two tissues jointly program β-cell adaption to irreversible impairment via cell cycle during week 4, organismal development around week 12, and immune cell trafficking at week 24. WGCNA and IPA also identified several possible interconnected molecules including GDF15. Alternations of T cell subpopulations validated the participation of adaptive immune response through a priming stage of decrement in anti-inflammatory Tregs and an amplification stage of increase in proinflammatory Th1 and Th17 cells in diabetic progression. Future in-depth research of individual gene will help to discover potential diagnostic and therapeutic targets for human T2DM.

### Limitations of the study

The limitations of our study are presented as follows: (1) although the standard diet from LabDiet (5001) is broadly used as control for the 60% fat diet from Research Diets (D12492) (Hafner et al., 2019; Kamimae-Lanning et al., 2015; Licholai et al., 2018; Sun et al., 2012; Wang et al., 2016), it is still not the perfect match due to ingredient differences in protein, carbohydrate, minerals, and vitamins, which could engender confounding factors for phenotypic and transcriptomic analyses.

(2) This study is powered to document longitudinal changes but the absolute timing of these changes may differ from the previous findings due to different substrains, sex, age of diet onset, and discriminatory capacity of each parameter. (3) The potential interconnected genes deduced from WGCNA and IPA can only suggest mathematical and possible biological correlation. To verify the causality, further thorough studies of individual gene using knockout mice and pathway inhibitors will be warranted to investigate its role in obesity-related diabetes. (4) Our HI/EG was performed without continuously infused [3-^3^H] glucose due to lab radiation use restrictions. Otherwise, the tissue-specific insulin resistance would have been more accurately assessed by radioactivity even in hypothalamus (Ono, 2019). (5) Due to the limited number of mice, our transcriptomic datasets lack of validation from proteomics or secretomics. (6) Our data cannot differentiate between changes in the composition of cells with tissues and those due to altered expression of genes in an individual population of cells.

### Resource availability

#### Lead contact

Further information and requests for resources and reagents should be directed to and will be fulfilled by the lead contact, Tao Yang (yangt@njmu.edu.cn).

#### Materials availability

This study did not generate new unique reagents.

#### Data and code availability

The transcriptomic datasets generated during this study are available at the Gene Expression Omnibus (GEO) repository under the accession number (GSE153222). All other data are available from the corresponding author upon request.

## Methods

All methods can be found in the accompanying transparent methods supplemental file.

## Supplemental information

Document S1. Transparent methods, Figures S1–S7, and Table S7

Table S1. List of islet DEGs related to KEGG or IPA pathways of T cell-mediated immunity, related to Figure 5 Genes without an asterisk (∗) are possible T cell-specific genes, and genes with an asterisk (∗) are possible genes related to T cell responses in non-lymphocytes.

Table S2. List of islet and liver modules, related to Figure 6 Information about number of genes inside each module, the most representative KEGG terms and if it is correlated to insulin secretion traits.

Table S3. The correlation matrix between modules and phenotypic traits, related to Figure 6 **(A) Heatmap showing the correlation matrix between islet modules and phenotypic traits,****r****elated to Figure 6** Each cell contains the corresponding Pearson coefficient and p-value. Highly positive or negative correlations are marked in red. **(B) Heatmap showing the correlation matrix between liver modules and phenotypic traits,****r****elated to Figure 6.** Each cell contains the corresponding Pearson coefficient and p-value. Highly positive or negative correlations are marked in red.

Table S4.Key genes found highly related to trait-correlated modules, related to Figure 6 **(A) List of islet key genes found highly related to trait-correlated liver modules,****r****elated to Figure 6.** Key genes that were highly representative of the module (membership>0.9) and highly correlated to a counterpart partner module (correlation>0.5) were identified between two trait-correlated islet and liver modules (Pearson coefficient >0.4). **(B) List of liver key genes found highly related to trait-correlated islet modules,****r****elated to Figure 6.** Key genes that were highly representative of the module (membership>0.9) and highly correlated to a counterpart partner module (correlation>0.5) were identified between two trait-correlated islet and liver modules (Pearson coefficient >0.4).

Table S5. Ingenuity network analysis of crosstalk between islets and liver, related to Figure 6 **(A) Ingenuity network analysis of crosstalk between islets and liver at 4 weeks of diet,****related to Figure 6.** Analysis was conducted using islet DEGs as the basis and sequentially adding liver DEGs encoded for proteins that can be secreted at 4 weeks of diet. The enriched networks are ranked by the scores. **(B) Ingenuity network analysis of crosstalk between islets and liver at 12 weeks of diet,****related to Figure 6.** Analysis was conducted using islet differentially expressed genes (DEGs) as the basis and sequentially adding liver DEGs encoded for proteins that can be secreted at 12 weeks of diet. The enriched networks are ranked by the scores. **(C) Ingenuity network analysis of crosstalk between islets and liver at 24 weeks of diet,****related to Figure 6.** Analysis was conducted using islet differentially expressed genes (DEGs) as the basis and sequentially adding liver DEGs encoded for proteins that can be secreted at 24 weeks of diet. The enriched networks are ranked by the scores.

Table S6. Summary results of sequencing reads per sample, related to transparent methods
