# Supplementary material for: Temporal metabolic and transcriptomic characteristics crossing islets and liver reveal dynamic pathophysiology in diet-induced diabetes
Source: iScience. 2021 Mar 5;24(4):102265. doi: 10.1016/j.isci.2021.102265 (PMC8008187; doi:10.1016/j.isci.2021.102265)
Supplement: Document S1. Transparent methods, Figures S1–S7, and Table S7 [file mmc1.pdf]

iScience, Volume *24*

Supplemental information

Temporal metabolic and transcriptomic characteristics crossing islets and liver reveal dynamic pathophysiology in diet-induced diabetes

Rui Gao, Qi Fu, He-Min Jiang, Min Shen, Rui-Ling Zhao, Yu Qian, Yun-Qiang He, Kuan-Feng Xu, Xin-Yu Xu, Heng Chen, Quan Zhang, and Tao Yang

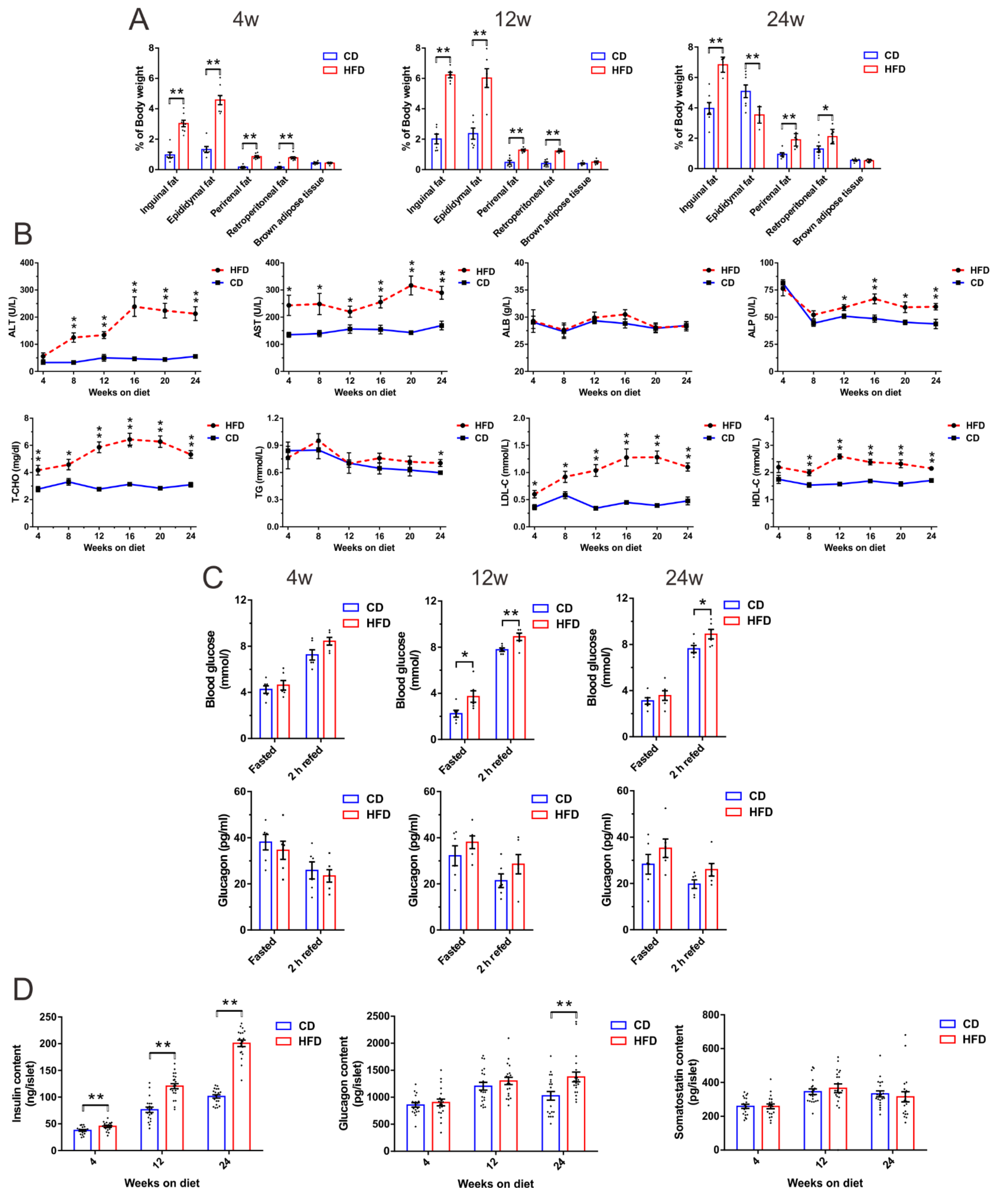

Figure S1. Fat distribution, biochemical tests, glucagon levels and hormone contents in CD and HFD mice, Related to Figure 1. (A) Analyses of inguinal, epididymal, perirenal, retroperitoneal fat depots and brown adipose tissue normalized to body weights (n=6−8 mice/group). **(B)** Biochemical tests of ALT, AST, ALP, ALB, T-CHO, TG, LDL-C and HDL-C after overnight fast (N=8 mice/group). **(C)** Blood glucose and plasma glucagon levels in fasted and 2 h refed HFD and CD mice after 4, 12 and 24 weeks of diet (N=6 mice/group). **(D)** Insulin, glucagon and somatostatin content in islets isolated from HFD and CD mice after 4, 12 and 24 weeks of diet (N=4−5 mice/group). All data are expressed as mean±SEM and analyzed using unpaired two-tailed *t*-test. **P*<0.05, ***P*<0.01.

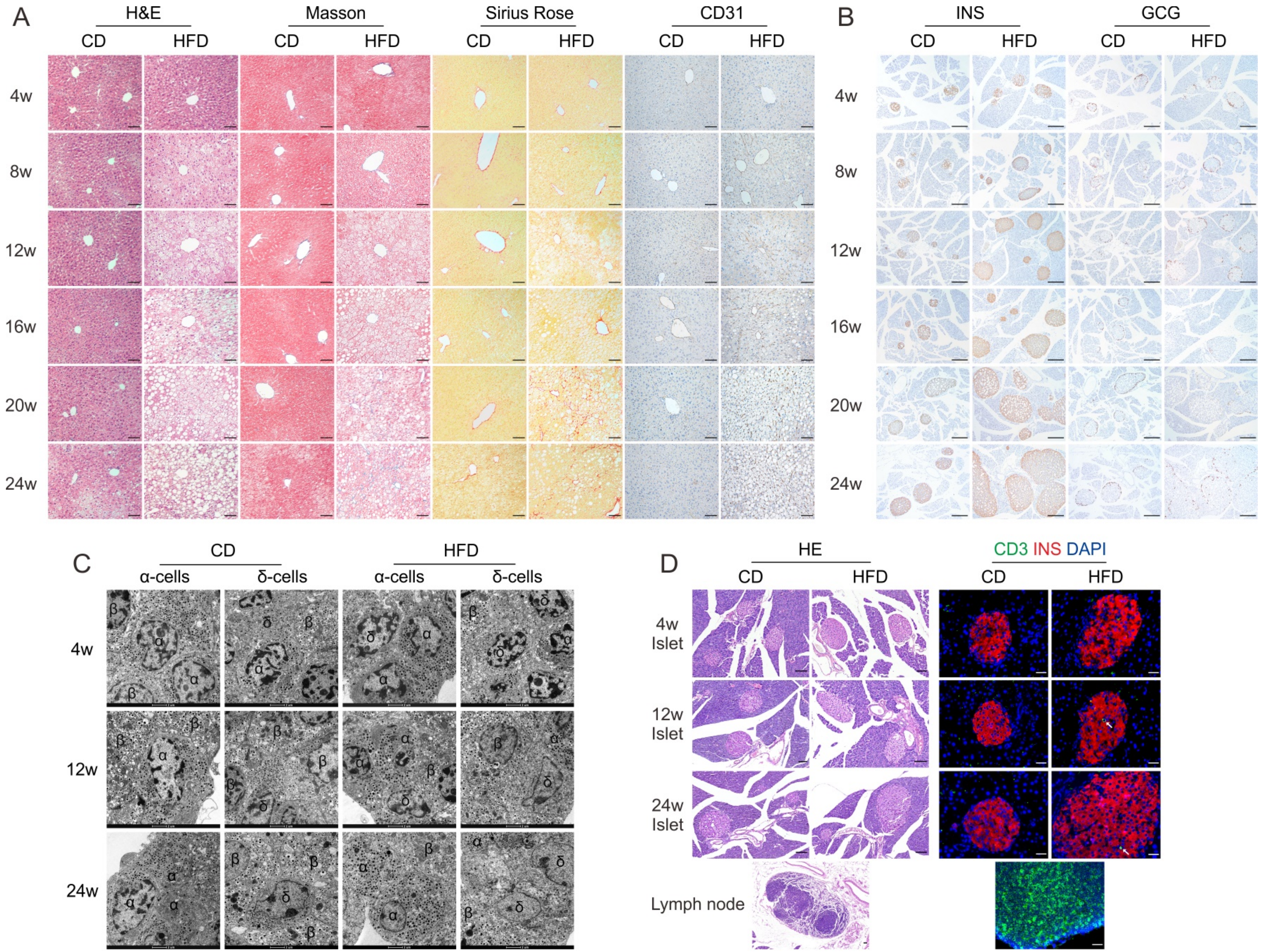

Figure S2. Impaired liver structure, enlarged islet mass, altered ultrastructure in α- and δ-cells, and evaluation of insulitis in HFD mice, Related to Figure 2. (A) Representative photomicrographs of liver sections stained with H&E for liver structure, Masson's Trichrome and Sirius Rose staining for collagen fiber deposition, and CD31 for microvessel density. Images are representative of 4 mice per group (scale bar: 20 μm). **(B)** Representitive immunohistochemical images showing consecutive pancreatic sections labeled either for insulin or glucagon. Images are representative of 4 mice per group (scale bar: 50 μm). **(C)** Representitive electron micrographs showing ultrastructural changes in α-cells and δ-cells. Images are representative of 8−12 α-cells or δ-cells/6−8 islets/3 mice per group (scale bar: 2 μm). **(D)** Representative H&E staining for evaluation of insulitis (left column, scale bar: 50 μm) and representative immunofluorescent images showing $CD3^+$ cells infiltrated islets in pancreatic sections (right column, scale bar: 20 μm). Images are representative of 4 mice per group.

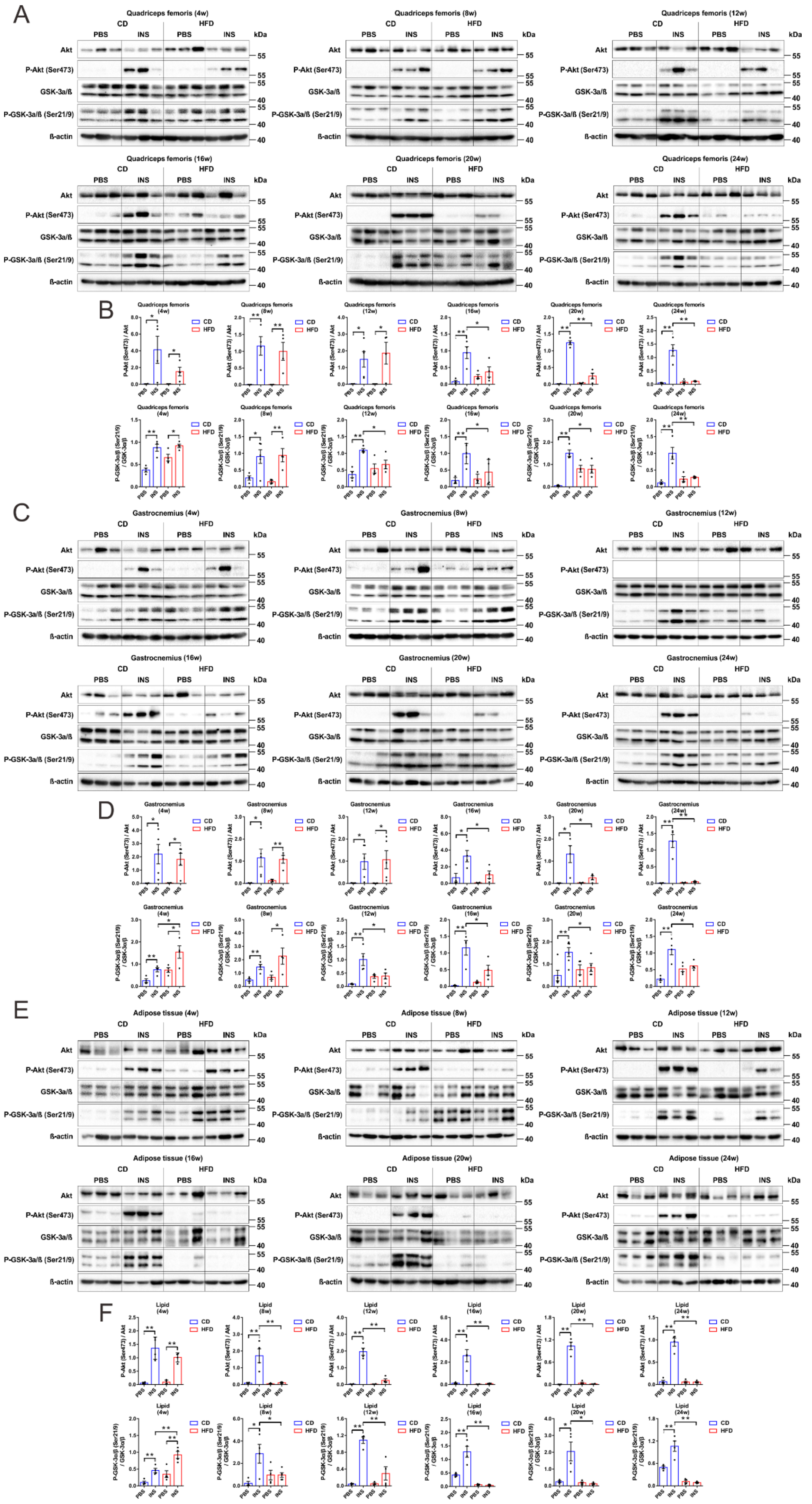

Figure S3. Longitudinal assessment of tissue-specific insulin sensitivity in quadriceps femoris, gastrocnemius and white adipose tissue, Related to Figure 3. (A, B) Immunoblot (A) and quantification (B) of Akt (Ser473) phosphorylation status relative to total Akt and GSK-3α/β (Ser21/9) phosphorylation status relative to total GSK-3α/β in quadriceps femoris (N=4−6 mice/group). **(C, D)** Immunoblot (C) and quantification (D) of these parameters in gastrocnemius (N=4−6 mice/group). **(E, F)** Immunoblot (E) and quantification (F) of these parameters in white adipose tissue (N=4−6 mice/group). All data are expressed as mean±SEM and analyzed using unpaired two-tailed *t*-test. **P*<0.05, ***P*<0.01.

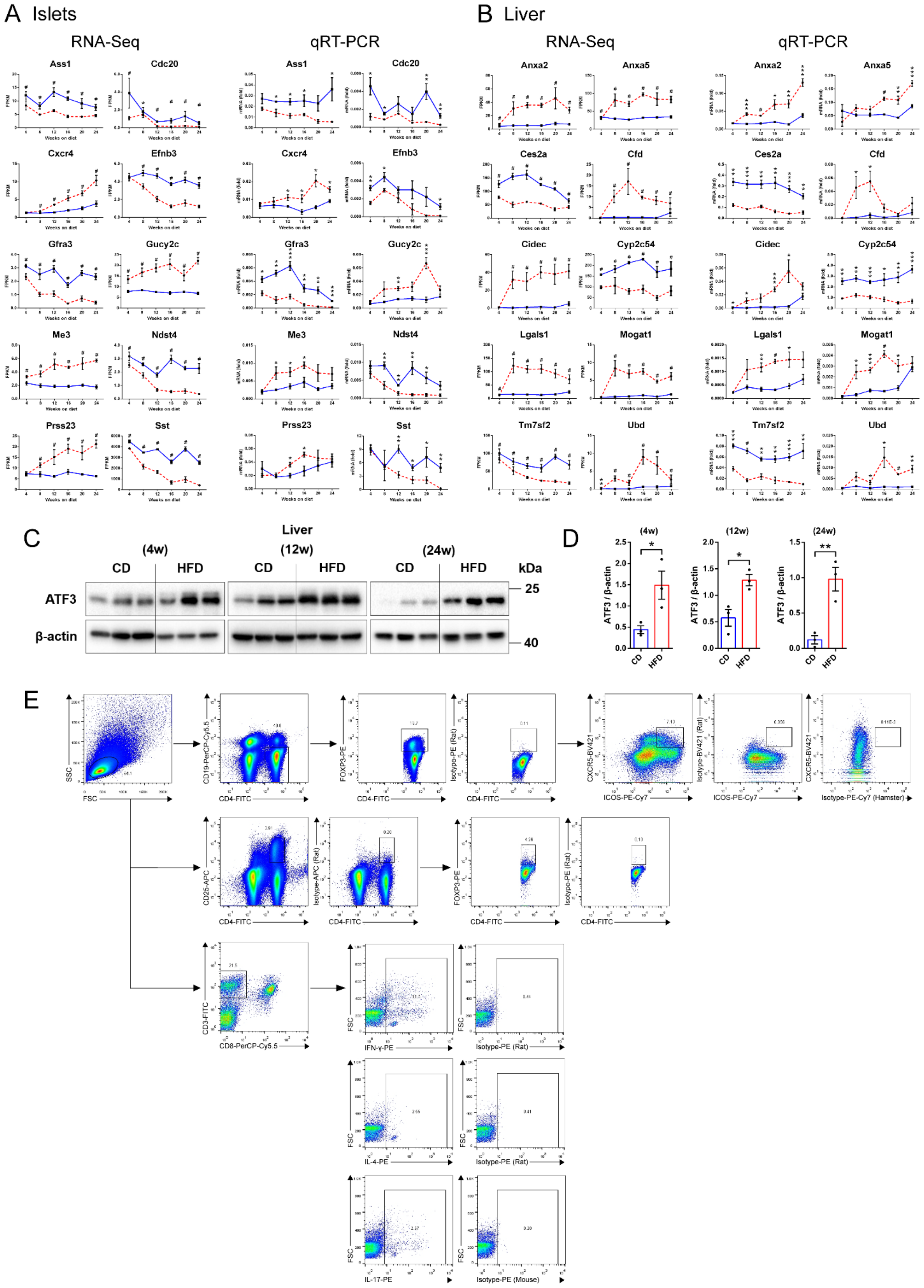

Figure S4. PCR and western blot validation of transcriptomic data, and gating strategies for flow cytometry, Related to Figure 4, 6 and 7. (A, B) Random selection of 10 DEGs for the validation of islet (A) and liver (B) transcriptomic data. The expression levels of these genes were measured in independent islet and liver samples (N=4 mice/group) (right column). The corresponding RNA-sequencing results of each gene were listed side by side for comparison (left column). **(C, D)** Immunoblot (C) and quantification (D) of ATF3 relative to β-actin in liver (N=3 mice/group). **(E)** Representative dot plots showing the gating strategies with respective isotype controls for flow cytometry. All data are expressed as mean±SEM and analyzed using unpaired two-tailed *t*-test. Left column of (A, B): *adjusted *P*<0.05, **adjusted *P*<0.01, ***adjusted *P*<0.001, #adjusted *P*<0.0001. Right column of (A, B): **P*<0.05, ***P*<0.01, ****P*<0.001, #*P*<0.0001.

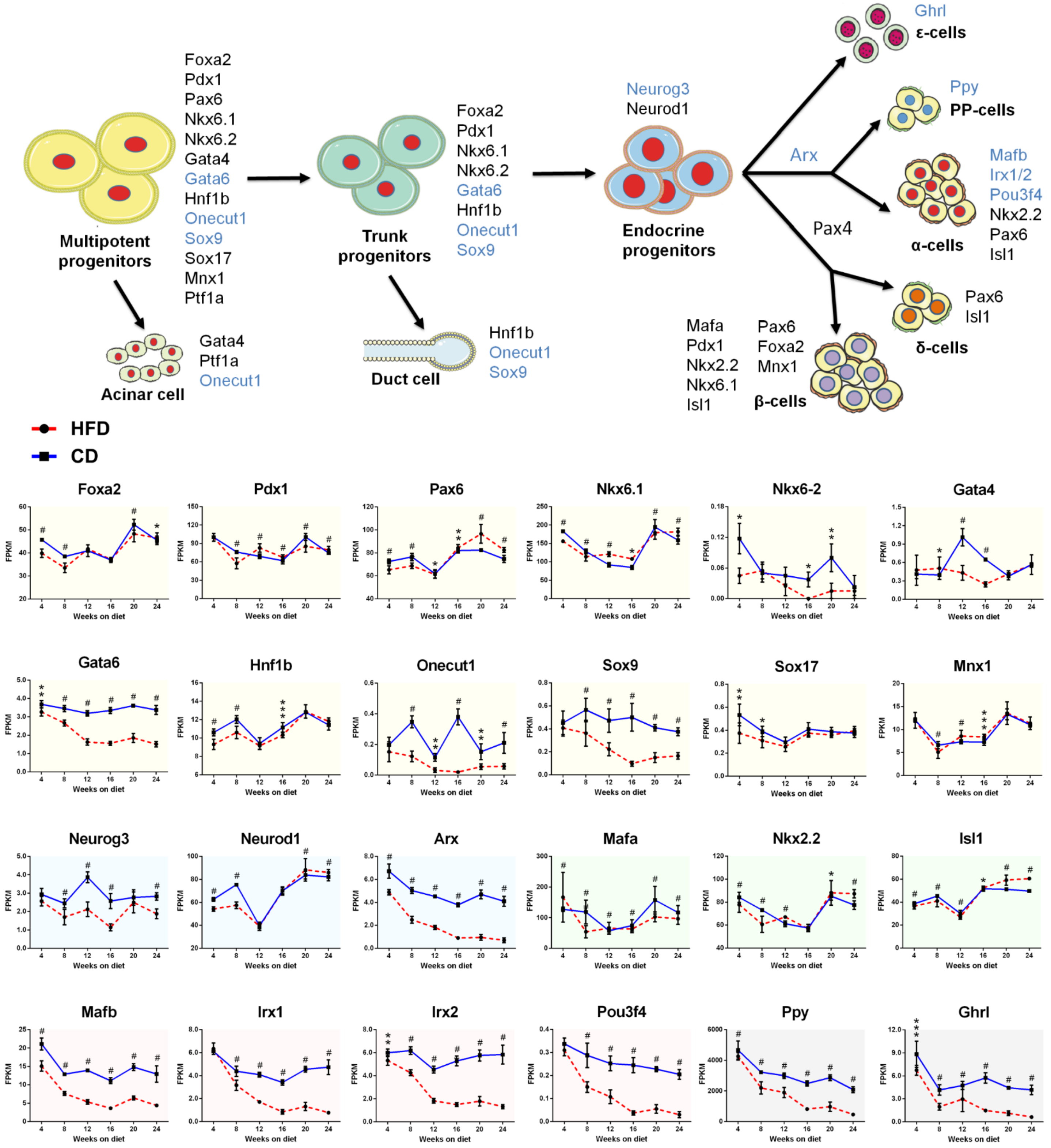

Figure S5. Time course expression of factors regulating endocrine pancreas development and β-cell mass, Related to Figure 4. The schematic representation depicted an overview of pancreatic progenitors toward differentiated lineages. Key transcription factors involved in each differentiation step are indicated and those significantly down-regulated are colored in blue. The time course expression data for these key genes are displayed in the line charts. Yellow, blue, green, red and grey shaded areas respectively refer to genes participating in the differentiation of multipotent progenitors, trunk progenitors, endocrine progenitors, hormone secreting α-, β-, δ-cells and PP-, ε-cells. All data are expressed as mean±SEM and analyzed using unpaired two-tailed *t*-test. *adjusted *P*<0.05, **adjusted *P*<0.01, ***adjusted *P*<0.001, #adjusted *P*<0.0001.

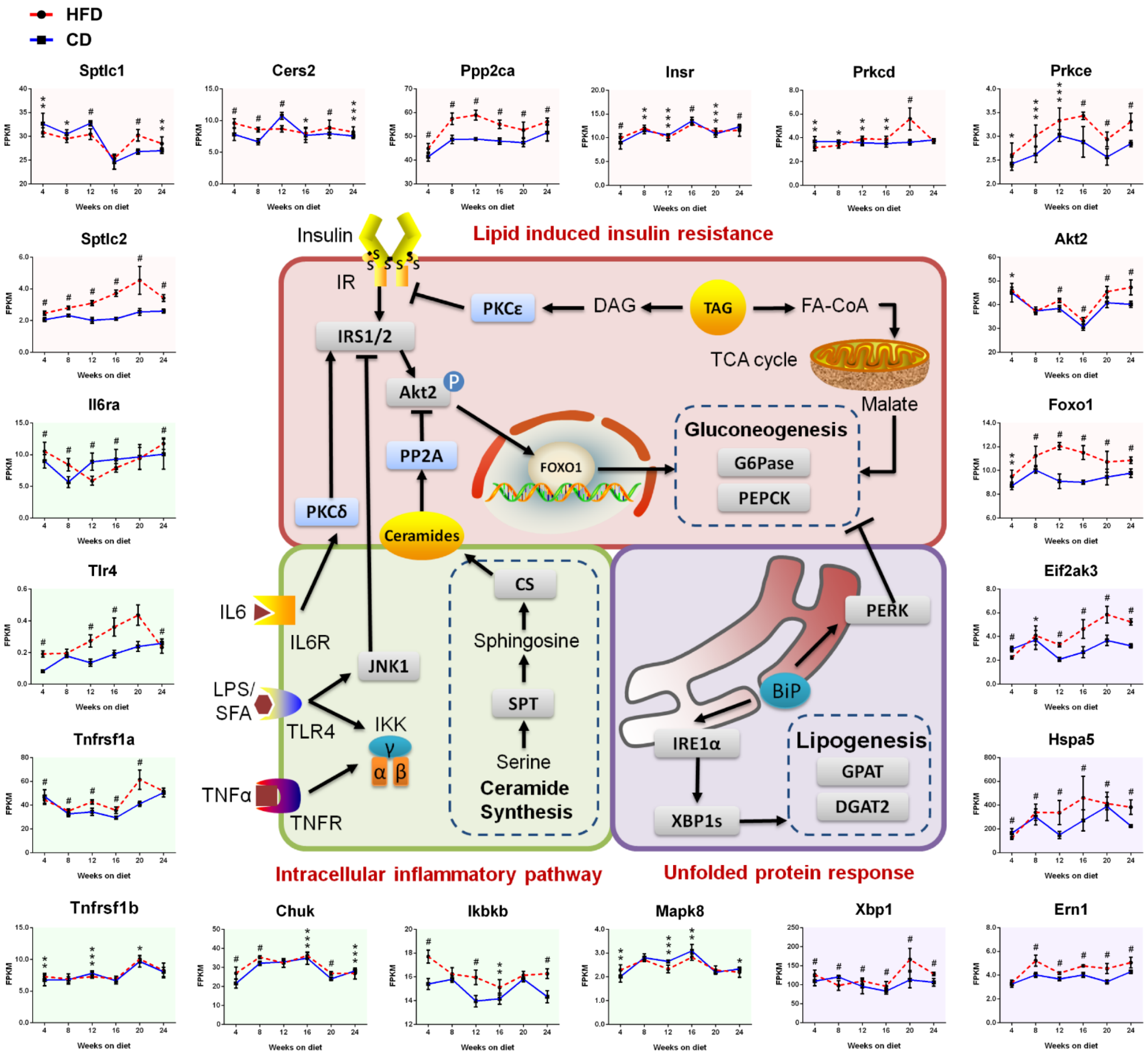

Figure S6. Time course expression of pathways involved in hepatic insulin resistance, Related to Figure 4. The schematic representation depicted three major mechanisms for hepatic insulin resistance. The time course expression data for these key genes are displayed in the line charts. Red, green and purple shaded areas respectively refer to genes participating in lipid-induced insulin resistance, intracellular inflammatory pathway and unfolded protein response. All data are expressed as mean±SEM and analyzed using unpaired two-tailed *t*-test. *adjusted *P*<0.05, **adjusted *P*<0.01, ***adjusted *P*<0.001, #adjusted *P*<0.0001.

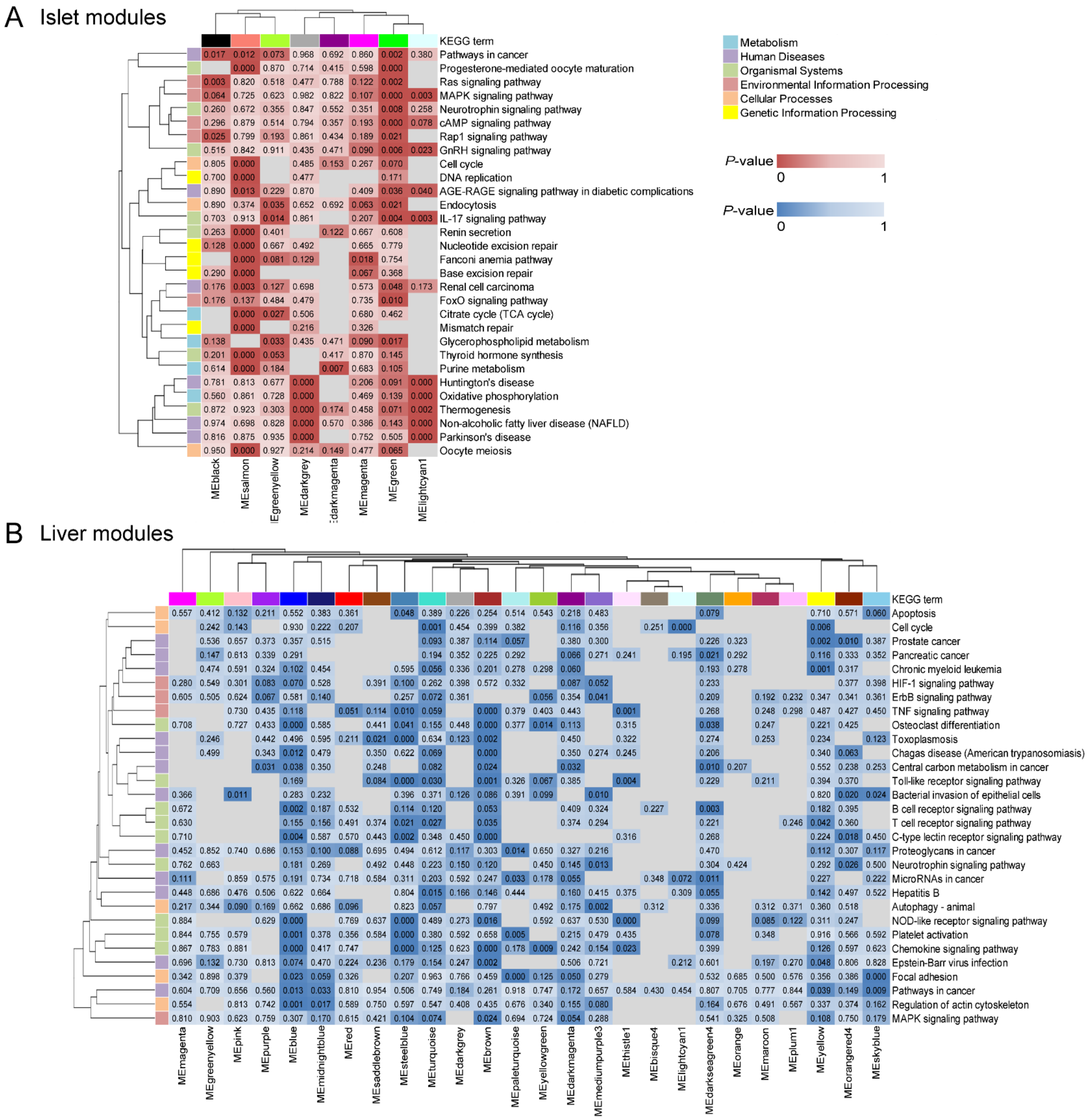

Figure S7. Pathway enrichment analyses of trait-correlated modules, Related to Figure 5 and 6. (A, B) Clustered heatmaps of islets (A) and liver (B) showing KEGG pathway enrichment analyses of trait-correlated modules. We displayed the overall top 30 over-represented pathways among these trait-correlated modules in islets and liver. Clustering by rows and columns was implemented using average linkage method. The intensity of color represents *P*-value.

Table S7. Primer sequences of quantitative RT-PCR for validation, Related to Transparent Methods.

Sample	Gene	5' Forward 3'	5' Reverse 3'
Islets	Ass1	GGTGCAGGTGTCTGTCTTCA	TCTGAAGGCGATGGTACTCC
Islets	Cdc20	TGGATCAAGGAGGGCAACTA	GCCCACATACTTCCTGGCTA
Islets	Cxcr4	GTGACTTCGAGAGCATCGTG	GTGGAGACGGAAGAGTGTCC
Islets	Efnb3	TGCCCTCACTACGAGAAGGT	TATAGCCAGGAGGAGCCAAA
Islets	Gfra3	CACCAAGCTACCTGTCTGGA	GAGGTCCGAGTCTGGTTTGA
Islets	Gucy2c	CGGATTGCCAGTTCCTGTAT	GCGCTAACGATCATGTCTGA
Islets	Me3	GGGAAGAAGCCACCTAAACC	GCTCAGGGCAAAGACGATAG
Islets	Ndst4	AGGGCACGAGAATGAATGTC	CTCAGCAGAAGGTCGTCACC
Islets	Prss23	TGACATCGGCATGGATTATG	CACAGAAGCGGTACACCAAA
Islets	Sst	GAGCCCAACCAGACAGAGAA	GAAGTTCTTGCAGCCAGCTT
Liver	Anxa2	TCATCTGCTCACGAACCAAC	ATCCTGGTCAATCAGCTCGT
Liver	Anxa5	CAGAGGATGTTGGTGGTCCT	TGTCCCAAAGATGGTGATGA
Liver	Ces2a	CCTGCAAGCACAGTTCAGAG	CCCACAAATAGGAGCCAAAG
Liver	Cfd	TGCACAGCTCCGTGTACTTC	CACCTGCACAGAGTCGTCAT
Liver	Cidec	GGACTTTATTGGCTGCCTGA	ATGTAGCTGGAGGTGCCAAG
Liver	Cyp2c54	CACTGTGGTGTTGCATGGAT	TCCTCTTGAACACGGTCCTC
Liver	Lgals1	CCAGCCTGTAGCCCTCAATA	CTCTCTGTGCTGGGCTCTCT
Liver	Mogat1	GCTGGCCTGTGTGGAAGTAT	AACAACGGGAAACAGAACCA
Liver	Tm7sf2	GCCTGGAACTGAAGGACAAG	GCTACCAAAGCCTTCGCATA
Liver	Ubd	ATGGCGGTTAATGACCTTTG	GCTTCACCACCTTCAGGGTA

Transparent Methods

Mice

Animal use and procedures were approved by the Medicine Animal Care Committee of Nanjing Medical University (IACUC1804001). All experiments were performed in accordance with the relevant institutional guidelines and regulations. Four-week-old C57BL/6N male mice were purchased from GemPharmatech Co., Ltd. and were maintained at 22°C on a 12 h light/dark cycle with *ad libitum* access to either CD (LabDiet 5001, 13.5% fat) or HFD (Research Diets D12492, 60% fat) for 4−24 weeks. Body weight, *ad libitum* blood glucose and food intake were monitored weekly. The average daily food intake was determined over 3 days by the net reduction in diet weight with the exclusion of spilled food. Energy intake is expressed as calories per mouse per day.

In vivo biochemical measurements

Serum insulin and glucagon levels were assessed using the Mouse Insulin and Glucagon ELISA kits (Mercodia, Uppsala, Sweden) according to the manufacturer’s instructions, respectively. For the measurement of glucagon, ethylenediaminetetraacetic acid and aprotinin were placed in sample collection tubes beforehand. For other biochemical tests, blood was collected from the retro-orbital sinus after overnight fast. Serum ALT, AST, ALP, ALB, T-CHO, TG, HDL-C and LDL-C levels after 16 h fasting were determined using an automatic biochemical analyzer (HITACHI 7100, Hitachi Koki Co. Ltd., Hitachinaka City, Japan).

Glucose and insulin tolerance tests

IPGTTs and ITTs were respectively performed after food withdrawal for 16 h or 5 h at intervals of 4 weeks. Tail blood glucose was measured (Medisafe Mini, Terumo, Japan) at baseline and periodically following intraperitoneal administration of 2 g/kg D-glucose for IPGTT or 0.75 U/kg insulin (Humulin R, Eli Lilly, Indianapolis, IN, USA) for ITT. At 5, 15, 30, 60 and 120 min after glucose injection during IPGTT, additional blood (25 μL) was collected via the tail vein for subsequent insulin determination.

Hyperinsulinemic-euglycemic clamps

Mice were subjected to carotid artery and jugular vein catheterization for sampling and infusion, respectively. The catheters were kept patent with heparin sodium (1 IU/mL) and body weights were recorded daily during recovery. After mice were fasted for 5 h, the clamp started at t=0 min (end of the 5 h fast) with a continuous insulin infusion (Humulin R, Eli Lilly, Indianapolis, IN) at a rate of 4 mU/kg/min. GIR was adjusted based on the measurement of blood glucose at 5 min intervals to maintain euglycemia at 6.1−7.2 mmol/L (110−130 mg/dL) during the steady-state period from t=80−120 min. Plasma insulin levels were determined from blood samples obtained from the carotid artery at t=-5 min prior to the insulin infusion and t=120 min. ISI is defined as ISI_{Clamp}=GIR/(GxΔI) where GIR is normalized for G (steady-state blood glucose concentration) and ΔI (difference between fasting and steady-state plasma insulin concentrations) (Loloi et al., 2018).

Purification of primary mouse islets

After clamped with a hemostat at the duodenal opening, the bile duct was cannulated and perfused with 2 mL collagenase P (Roche Diagnostics, Germany, 1 mg/mL in Hanks' balanced salt solution (HBSS)). Pancreatic tissue was digested at 37 °C for 10 min. The reaction was stopped by adding cold HBSS with 10% fetal bovine serum (FBS), followed by 10 seconds of vigorous shaking. Islets were separated onto a gradient composed of HBSS and Histopaque (11191 and 10771, Sigma Aldrich, USA) layers and were hand-picked for 2 times under a dissection microscope to minimize acinar cells. The purity of islets for RNA-sequencing was evaluated by the expression of *Amy1a* (a marker of acinar cells) and *Krt19* (a marker of ductal cells). In our transcriptomic dataset, *Amy1a* and *Krt19* showed no statistical difference at mRNA level from any time points, which indicates the contaminants were fairly minimized.

In vitro hormone secretion measurements

Islets were isolated and pooled from 4−5 mice per group, then incubated overnight in RPMI-1640 supplemented with 10% FBS and 1% Pen/Strep. The next day, groups of 20 size-matched islets were pre-incubated for 1 h in Krebs-Ringers-Bicarbonate-HEPES (KRBH) buffer, containing (in mmol/L): 140 NaCl, 3.6 KCl, 2 $NaHCO_3$, 0.5 NaH_2PO_4, 0.5 $MgSO_4$, 5 HEPES, 2.6 $CaCl_2$, 1

glucose and 0.2% (wt/vol.) bovine serum albumin (BSA) (pH 7.4 with NaOH). Following this, islets were sequentially subjected to KRBH solutions containing 2 or 20 mmol/L glucose with or without 0.5 mM PA for 1 h at 37°C in 5% CO_2. PA was non-covalently conjugated to fatty acid-free BSA at a 6.6:1 molar ratio. Supernatants were collected and islets were lysed overnight in acid ethanol buffer (75% EtOH, 0.55% HCl). Insulin (Mercodia, Uppsala, Sweden), glucagon (Mercodia, Uppsala, Sweden) and somatostatin (Phoenix Pharmaceuticals, CA, USA) in supernatants and contents were determined using the ELISA kits following the manufacturer's instructions. Secreted hormones were calculated as percentage of total contents per hour.

Electron microscopy

For TEM, isolated islets from 3 mice per group were pooled and cultured overnight. After pre-incubation and treatment with 2 or 20 mM glucose in KRBH buffer for 1 h at 37°C, islets were fixed in 2.5% glutaraldehyde and 2.5% paraformaldehyde in cacodylate buffer (0.1 M, pH 7.4). Then, samples were post-fixed in 1.0% osmium tetroxide for 2 h and stained with 2% (wt/vol.) uranyl acetate in double distilled water to increase the contrast. After washing and dehydration through graded alcohol (50, 70, 90 and 100%), samples were embedded in EPON 812, and ultrathin sections (60−80 nm thick) were cut by a diamond knife using a Leica ultra-microtome EM UC6 (Leica, Wetzlar, Germany). Islets were examined by FEI Tecnai G2 Spirit Bio TWIN (FEI, Hillsboro, USA) with an accelerating voltage of 80 kV. Mature insulin granules were determined and quantified by grayscale threshold analyses. Immature granules, as well as docked granules, were identified and counted manually. Granules located within 100 nm from the plasma membrane and without signs of fusion were considered docked (Masini et al., 2012).

Histopathological and immunohistochemical staining

After the mice were transcardically perfused with 4% (wt/vol.) paraformaldehyde, pancreas and livers were dissected from each group, fixed in 4% paraformaldehyde and dehydrated in a graded sucrose series. The paraffin-embedded pancreatic samples were then continuously sectioned at 5 μm, rehydrated with a sequential wash in xylene, 100%, 95%, 75% ethanol and water, and then boiled in 1 mM ethylenediaminetetraacetic acid for antigen retrieval. Hematoxylin and eosin (H&E)

staining was conducted for the evaluation of peri-insulitis (Solarbio, Beijing, China). For immunofluorescent staining, after blocking with 5% goat serum, slides were incubated overnight with primary antibodies (anti-insulin (ab7842, 1:400), anti-glucagon (ab10988, 1:400), anti-somatostatin (ab30788, 1:100), anti-Ki67 (ab15580, 1:1000) (Abcam, Cambridge, UK) and anti-CD3 (17617-1-AP, 1:100) (Proteintech, IL, USA)). The next day, sections were washed, incubated with conjugated or biotinylated secondary antibodies for 1 h at room temperature, washed again and sealed with ProLong™ Gold Antifade Mountant with or without DAPI (Thermo Scientific, Waltham, MA, USA). The paraffin-embedded liver tissues were also sliced at 5 μm, and stained with H&E for liver structure, Masson's Trichrome and Sirius Rose for collagen fiber deposition (Solarbio, Beijing, China). Primary antibody anti-CD31 (ab28364, 1:50) (Abcam, Cambridge, UK) was used for immunostaining to assess hepatic microvessel density. Images were captured using Axiovert A1 Inverted Microscope (Carl Zeiss, Jena, Germany).

Western blot

Liver, quadriceps femoris, gastrocnemius and white adipose tissue were harvested 10 min after an intraperitoneal bolus of insulin (5 U/kg) or PBS from overnight fasted mice. After homogenized in RIPA buffer (R0278, Sigma Aldrich, USA) with 1:100 dilution of protease inhibitor cocktail (P8340, Sigma Aldrich, USA), tissue extracts were centrifuged at high speed (15,000 g) to eliminate insoluble material. Protein concentrations in the supernatants were measured using the bicinchoninic acid assay (Pierce BCA Protein Assay Reagent, Thermo Scientific, Waltham, MA, USA). Equal amount of protein lysates (40 μg) was separated by SDS-polyacrylamide gel electrophoresis (SDS-PAGE) and then transferred to PVDF membranes (Millipore, Burlington, MA, USA). The resulting blots were blocked with 5% BSA in TBST for 1 h at room temperature and incubated with primary antibodies overnight at 4°C (anti-phospho-Akt (Ser473) (4060, 1:1000), anti-Akt (4691, 1:1000), anti-phospho-GSK-3α/β (Ser21/9) (8566, 1:1000), anti-GSK-3α/β (5676, 1:1000), anti-β-Actin (13E5, 1:1000) (Cell Signaling, Danvers, MA, USA), and anti-ATF3 (sc-188, 1:1000) (Santa Cruz Biotechnology, Dallas, TX, USA)). After washed for three times with TBST, incubated with HRP-conjugated secondary antibodies for 1 h at room temperature and washed for three times again, SuperSignal Chemiluminescent Substrates (Thermo Scientific, Waltham, MA,

USA) were applied for detection. Values were expressed as phospho-protein over total protein.

Flow cytometry

Anti-mouse CD3 fluorescein isothiocyanate (FITC) (100306), anti-mouse CD4 FITC (100510), anti-mouse CD8 peridinin-chlorophyll protein (PerCP)-cyanine 5.5 (Cy5.5) (100734), anti-mouse CD19 PerCP-Cy5.5 (115534), anti-mouse CD25 allophycocyanin (APC) (101910), anti-mouse inducible costimulator (ICOS) phycoerythrin (PE)-cyanine 7 (Cy7) (313520), anti-mouse C-X-C motif chemokine receptor 5 (CXCR5) Brilliant Violet 421™ (145511), anti-mouse interferon (IFN)-γ PE (505808), anti-mouse IL-4 PE (504104), anti-mouse IL-17 PE (517008), and the respective isotype controls including PE rat IgG1 κ isotype control (505807), PE mouse IgG1 κ isotype control (400111), APC rat IgG2b κ isotype control (400611), PE/Cyanine7 hamster IgG isotype control (400921) and Brilliant Violet 421™ rat IgG2b κ isotype control (400655) (BioLegend, San Diego, CA, USA) were used. For forkhead box P3 (FOXP3) intracellular staining, anti-mouse FOXP3 PE, FOXP3 Fix/Perm Kit and PE rat IgG2a κ isotype control (eBioscience, San Diego, CA, USA) were used according to the manufacturer's instructions.

Mouse single cell suspensions of spleens, draining lymph nodes (including peri-pancreas lymph nodes) were prepared. Cells were first stained with surface antibodies for 30 min at 4°C in the dark. After surface staining, cells were fixed, permeabilized for 40 min in 1×FOXP3 Fix/Perm Buffer and washed twice with 1× Perm Buffer. Cells were then incubated with FOXP3 antibody for 30 min. For intracellular cytokines detection, cells were stimulated with phorbol myristate acetate (PMA) and ionomycin in the presence of brefeldin A (BioLegend, San Diego, CA, USA) for 5 h before surface staining. Cells were then fixed with Intracellular Fixation Buffer (BD Biosciences, San Diego, CA, USA), incubated with Permeabilization Buffer (BD Biosciences, San Diego, CA, USA), and then stained with anti-mouse IFN-γ PE, anti-mouse IL-4 PE or anti-mouse IL-17 PE. The cytometric data were collected using a FACS Aria II Sorp flow cytometer (BD Biosciences, San Diego, CA, USA).

RNA extraction, library construction and sequencing

Total RNA was isolated from handpicked islets and homogenized liver tissue using TRIzol reagent

(Invitrogen, CA, USA) in accordance with the manufacturer's instructions. The quality and quantity of extracted RNA were measured by on-chip electrophoresis utilizing the Agilent RNA 6000 Nano Kit and Agilent 2100 Bioanalyzer (Agilent Technologies, CA, USA). Samples exhibited 1.9≤A260/A280≤2.2, RNA integrity number>8.0 and 28S/18S>1.0 were selected for library preparation. The total RNA was purified by enriching poly (A) mRNA with magnetic oligo (dT) beads. After reverse transcription by using the random hexamer (N6) primers, double-stranded cDNA fragments were synthesized and subjected to end repair and adapter ligation. The cDNA libraries were then constructed by PCR amplification, and finally sequenced on BGISEQ-500 sequencing platform by the Beijing Genomics Institute (Shenzhen, China).

Reads filtering and de novo assembly

The sequencing raw reads were filtered for low-quality, adaptor-polluted, high content of unknown base reads by SOAPnuke (v1.5.2) (Cock et al., 2010). We used Trinity (v2.0.6) (Grabherr et al., 2011) to perform de novo assembly, and Tgicl (v2.0.6) (Pertea et al., 2003) on cluster transcripts to remove redundancy and get unigenes. The high-quality clean reads were then mapped to the mouse reference genome (GRCm38) via HISAT2 (v2.0.4) (Kim et al., 2015) and full-length transcriptomic database via Bowtie2 (v2.2.5) (Langmead and Salzberg, 2012) (**Table S6**). The gene expression levels were then quantified by RSEM (v1.1.12) (Li and Dewey, 2011) and were normalized by the method of fragments per kilobase of exon model per million reads mapped (FPKM). The DEseq2 (fold change≥2 and adjusted *P*-value≤0.05) package was applied to identify DEGs between HFD and CD groups at each time point.

Pathway analyses

To interpret the functional significance of DEGs, IPA (Qiagen, Redwood City, CA, USA, www.qiagen.com/ingenuity) was conducted to determine enriched canonical pathways, upstream regulators, mechanistic networks, and diseases and functions. DEGs with a $\log_2$fold change ($\log_2$FC) either ≤−1 for down-regulated or ≥1 for up-regulated, adjusted *P*-value ≤0.05 and average FPKM >1 were selected. KEGG analyses of DEGs were also performed according to the gene annotations provided by DAVID online tool (v6.8). Pathway significance is expressed as

P-value calculated by right-tailed Fisher's exact test, which indicates the possibility that the correlation between DEGs from our dataset and a given process/function is due to random chance.

Secretome data were obtained from Vertebrate Secretome Database (VerSeDa) (Cortazar et al., 2017), which stores information about proteins that are predicted to be secreted through the classical and non-classical mechanisms, for a wide range of vertebrate species deposited at NCBI, UCSC and ENSEMBL sites.

Weighted gene co-expression networks between islets, liver and metabolic traits

We separately conducted WGCNA for 48 islet and 48 liver samples. Gene modules (clusters of genes displaying similar correlated patterns of transcription) were built by employing the WGCNA package in R (Langfelder and Horvath, 2008), following the general guidelines (Zhang and Horvath, 2005). Two inclusion criteria for filtering genes in WGCNA construction were applied: (1) genes with FPKM>0 in any of the 48 samples were selected; (2) median absolute deviation (MAD) was calculated for each gene as a robust measure of variability, and the 75% most varying genes were chosen. Briefly, pairwise Pearson correlation coefficients between all included genes were calculated to generate a signed similarity. After a sensitivity analysis of scale-free topology (R^2>0.9), soft threshold power was respectively set to 6 for islets and 18 for liver to obtain a weighted adjacency matrix. Then it converted into a topological overlapping matrix network which was used as input for a hierarchical clustering analysis. The modules were finally identified by implementing the dynamic hierarchical tree-cut algorithm, using the parameters deepSplit=2 and minClusterSize=20. Module eigengenes which were defined as the principal components of each module were calculated using the moduleEigengenes function.

We associated the islet and liver modules by measuring the Pearson correlation using the WGCNA "relating modules to external information" analyses. The significance (Pearson correlation) was calculated for each gene in islet modules to the corresponding correlated liver modules, and for each gene in liver modules to the corresponding correlated islet modules. We also obtained the connectivity (membership) of each gene to its own module. Pairwise comparisons of all modules could identify a set of 'key genes' which were most correlated to the

modules of other tissue (>0.5) and also displayed a high membership in its own module (>0.9).

To identify gene modules associated with traits such as fasting glucose level, we also performed the same analyses ("relating modules to external information") to correlate islet and liver modules to metabolic variables (weight, energy intake, fasting glucose, fasting insulin and other derivative parameters from IPGTT and ITT, ALT, AST, ALP, ALB, T-CHO, HDL-C, LDL-C and TG, listed in **Table S3**). The cut-off of *P*-value for selecting modules of interest was set to 0.05. Modules of interest were further characterized by KEGG pathway enrichment analyses.

Quantitative real-time PCR

To validate the reliability of data obtained by RNA-sequencing, qRT-PCR was performed. Total RNA was extracted and its quality and quantity were examined as indicated above. 1 μg of total RNA was reverse transcribed using Takara PrimeScript™ RT Master Mix (Clontech Laboratories, USA). qRT-PCR was performed on Step One Plus Real-Time PCR System (Applied Biosystems, USA) with SYBR Premix Ex Taq II Kit (Clontech Laboratories, USA) and primers presented in **Table S7**. β-Actin was served as an internal control and relative changes in mRNA expression were calculated by the comparative ΔCt method (Livak and Schmittgen, 2001).

Quantification and statistical analysis

The images presented in the manuscript are representative of the data and the image/staining quality. The area occupied by glucagon or somatostatin staining (area A) was measured using Image J and expressed relative to that of insulin staining (area B) by the formula A / (A+B). All values are expressed as mean±SEM and compared by Student's unpaired *t*-test or analysis of variance (ANOVA) with Tukey's test which corrects for multiple hypotheses. Correlation was quantified by the Pearson's r for parametric data analysis. AUC was calculated by trapezoid analysis and was compared by *t*-test. A two-sided *P*-value<0.05 was considered statistically significant. Graphs and statistical analyses were completed using GraphPad Prism 6.0 (GraphPad Software, San Diego, CA, USA).

Supplemental References

Cock, P.J., Fields, C.J., Goto, N., Heuer, M.L., and Rice, P.M. (2010). The Sanger FASTQ file format for sequences with quality scores, and the Solexa/Illumina FASTQ variants. Nucleic Acids Res *38*, 1767-1771.

Cortazar, A.R., Oguiza, J.A., Aransay, A.M., and Lavin, J.L. (2017). VerSeDa: vertebrate secretome database. Database (Oxford) *2017*.

Grabherr, M.G., Haas, B.J., Yassour, M., Levin, J.Z., Thompson, D.A., Amit, I., Adiconis, X., Fan, L., Raychowdhury, R., Zeng, Q., et al. (2011). Full-length transcriptome assembly from RNA-Seq data without a reference genome. Nat Biotechnol *29*, 644-652.

Kim, D., Langmead, B., and Salzberg, S.L. (2015). HISAT: a fast spliced aligner with low memory requirements. Nat Methods *12*, 357-360.

Langfelder, P., and Horvath, S. (2008). WGCNA: an R package for weighted correlation network analysis. BMC Bioinformatics *9*, 559.

Langmead, B., and Salzberg, S.L. (2012). Fast gapped-read alignment with Bowtie 2. Nat Methods *9*, 357-359.

Li, B., and Dewey, C.N. (2011). RSEM: accurate transcript quantification from RNA-Seq data with or without a reference genome. BMC Bioinformatics *12*, 323.

Livak, K.J., and Schmittgen, T.D. (2001). Analysis of relative gene expression data using real-time quantitative PCR and the 2(-Delta Delta C(T)) Method. Methods *25*, 402-408.

Loloi, J., Miller, A.J., Bingaman, S.S., Silberman, Y., and Arnold, A.C. (2018). Angiotensin-(1-7) contributes to insulin-sensitizing effects of angiotensin-converting enzyme inhibition in obese mice. Am J Physiol Endocrinol Metab *315*, E1204-E1211.

Masini, M., Marselli, L., Bugliani, M., Martino, L., Masiello, P., Marchetti, P., and De Tata, V. (2012). Ultrastructural morphometric analysis of insulin secretory granules in human type 2 diabetes. Acta Diabetol *49 Suppl 1*, S247-252.

Pertea, G., Huang, X., Liang, F., Antonescu, V., Sultana, R., Karamycheva, S., Lee, Y., White, J., Cheung, F., Parvizi, B., et al. (2003). TIGR Gene Indices clustering tools (TGICL): a software system for fast clustering of large EST datasets. Bioinformatics *19*, 651-652.

Zhang, B., and Horvath, S. (2005). A general framework for weighted gene co-expression network

analysis. Stat Appl Genet Mol Biol *4*, Article17.
